# Characterization of the Tau Interactome in Human Brain Reveals Isoform-Dependent Interaction with 14-3-3 Family Proteins

**DOI:** 10.1523/ENEURO.0503-22.2023

**Published:** 2023-03-21

**Authors:** Ryan K. Betters, Emma Luhmann, Amy C. Gottschalk, Zhen Xu, Mallory R. Shin, Christopher P. Ptak, Kimberly L. Fiock, Lilliana C. Radoshevich, Marco M. Hefti

**Affiliations:** 1 Department of Pathology; 2 Interdisciplinary Neuroscience Graduate Program; 3 Department of Microbiology and Immunology; 4 College of Liberal Arts and Sciences; 5 Protein and Crystallography Facility; 6 Nuclear Magnetic Resonance Core Facility; 7 Iowa Neuroscience Institute, University of Iowa, Iowa City, IA

**Keywords:** 14-3-3, development, interactome, resilience, tau

## Abstract

Despite exhibiting tau phosphorylation similar to Alzheimer’s disease (AD), the human fetal brain is remarkably resilient to tau aggregation and toxicity. To identify potential mechanisms for this resilience, we used co-immunoprecipitation (co-IP) with mass spectrometry to characterize the tau interactome in human fetal, adult, and Alzheimer’s disease brains. We found significant differences between the tau interactome in fetal and AD brain tissue, with little difference between adult and AD, although these findings are limited by the low throughput and small sample size of these experiments. Differentially interacting proteins were enriched for 14-3-3 domains, and we found that the 14-3-3-β, η, and γ isoforms interacted with phosphorylated tau in Alzheimer’s disease but not the fetal brain. Since long isoform (4R) tau is only seen in the adult brain and this is one of the major differences between fetal and AD tau, we tested the ability of our strongest hit (14-3-3-β) to interact with 3R and 4R tau using co-immunoprecipitation, mass photometry, and nuclear magnetic resonance (NMR). We found that 14-3-3-β interacts preferentially with phosphorylated 4R tau, forming a complex consisting of two 14-3-3-β molecules to one tau. By NMR, we mapped 14-3-3 binding regions on tau that span the second microtubule binding repeat, which is unique to 4R tau. Our findings suggest that there are isoform-driven differences between the phospho-tau interactome in fetal and Alzheimer’s disease brain, including differences in interaction with the critical 14-3-3 family of protein chaperones, which may explain, in part, the resilience of fetal brain to tau toxicity.

## Significance Statement

Aggregation of phosphorylated tau is the final common pathway for neuronal death across multiple neurodegenerative diseases, but the mechanisms that trigger this process remain unclear. The fetal brain shows high levels of potentially toxic phosphorylated tau without apparent adverse effects, and therefore represents an unprecedented, and heretofore unexplored, opportunity to identify age-related mechanisms for tau toxicity. Our results use a novel dataset mapping the tau interactome in human fetal, adult, and Alzheimer’s disease (AD) brain to identify splicing-dependent changes in tau interaction with 14-3-3 family chaperone proteins, which represent highly plausible protective mechanisms in the fetal brain.

## Introduction

Aggregation of phosphorylated tau protein acts as the final common pathway for neurotoxicity across multiple neurodegenerative diseases, including frontotemporal lobar degeneration with tau (FTLD-tau), chronic traumatic encephalopathy (CTE), and Alzheimer’s disease (AD). Human fetal tau is extensively phosphorylated, with a pattern similar to that in Alzheimer’s disease, but without apparent adverse effects (Yu et al., 2009; Hefti et al., 2019). The reasons for this remarkable resilience to tau toxicity remain unclear. One key difference is that tau with four microtubule binding repeats (4R) is seen only in the adult brain, while fetal tau consists exclusively of the shorter (3R) isoform (Andreadis, 2005). Mixed tauopathies such as AD and 4R tauopathies such as progressive supranuclear palsy (PSP) or corticobasal degeneration (CBD) are relatively common. Interestingly however, Pick’s disease, the only known 3R tauopathy, is extremely rare (Gotz et al., 2019). Tau splicing is thought to change affinity to multiple tau binding partners, but its protective role in the developing brain has not been explored (Panda et al., 2003; Best et al., 2019). In the current report, we characterize the fetal, adult, and Alzheimer tau protein interactome and identify tau splicing-dependent changes in tau-14-3-3 protein interaction as a potential protective mechanism in the fetal brain.

## Materials and Methods

### Human brain tissue procurement

Frozen cortical adult and fetal human brain tissue was obtained from the brain bank or the NIH NeuroBioBank. Inclusion criteria were postmortem interval <24 h and an appropriate neuropathological diagnosis (Alzheimer’s disease, normal control) according to published criteria (Montine et al., 2012). Exclusion criteria were gestational age <18 or >22 postconceptional weeks or any elective termination as defined by state and federal law. Cases with pathologic evidence of global hypoxic ischemic injury were excluded. This research was reviewed by the University of Iowa Institutional Review Board and determined not constitute human subjects research under the NIH Revised Common Rule. Age, gender, source, and diagnosis of the cases used are shown in Table 1.

**Table 1:** Demographics of cases used in study

Case ID	Age	Sex	Brain diagnosis	Source	Cause of death	**BBID**
Fetal 1	0	F	None	NIH	Prematurity	4510
Fetal 2	0	F	None	NIH	Prematurity	1756
Fetal 3	0	M	None	NIH	Prematurity	1085
Fetal 4	0	M	None	NIH	Prematurity	4711
Adult 1	66	F	None	University of Iowa	Interstitial lung disease	P0002
Adult 2	58	M	None	University of Iowa	Cardiovascular disease	P0005
Adult 3	60	F	None	University of Iowa	Overdose	P0006
AD 1	59	M	AD	University of Iowa	c/o AD	P0036
AD 2	65	M	AD	University of Iowa	c/o AD	P0053
AD 3	67	F	AD	University of Iowa	c/o AD	P0062

### Homogenization and preparation of human brain samples

Frozen brain tissue stored at −8°C was mechanically pulverized using a stainless-steel mortar and pestle on dry ice. We then added 600 μL of ice-cold lysis buffer [20 mm Tris, 1 mm EDTA, 1 mm EGTA, 240 mm sucrose, 1× Halt Protease and Phosphatase Inhibitor (ThermoFisher)] to 1.5-ml tubes containing ceramic beads (#15-340-153, Fisherbrand), followed by 100 mg of pulverized frozen brain. We homogenized the samples using a Bead Mill 4 homogenizer (Fisherbrand) with velocity 5 m/s for a total of 20 s. We removed the supernatant and transferred it into a sterile low-protein binding microcentrifuge tube, spun at 15,000 × *g* for 30 min at 4 C, and the supernatant then discarded. We measured final protein concentrations in the pellet using a BCA (bicinchoninic acid) assay before proceeding directly to immunoprecipitation (IP) or storage at −80° C. Samples were prepared for co-immunoprecipitation through treatment with Benzonase endonuclease (#9025-65-4, Millipore) for 1 μg per reaction and 15-min room temperature immediately before co-IP. We used benzonase because in our pilot study, samples without benzonase were extremely viscous, raising concerns for nonspecific interactions in the resulting mass spectrometry data (data not shown).

### Antibodies

All primary and secondary antibodies, including resource IDs, are listed with the concentrations used in Table 2.

**Table 2:** Antibodies used for study

Name	Catalog #	Manufacturer	RRID	Concentration
HT7	MN1000	Invitrogen	RRID:AB_2314654	1:500 (WB), 5 μg/500 μg (co-IP)
ANTI-FLAG M2	F1804	Sigma-Aldrich	RRID:AB_262044	10 μg/500 μg beads (co-IP)
14-3-3-β	Ab15260	Abcam	RRID:AB_301799	1:1000 (WB)
p-Ser/Thr	9631	Cell Signaling	RRID:AB_330308	1:1000 (dot blot)
pSer214-tau	Ab170892	Abcam	RRID:AB_2905610	1:1000 (dot blot)
Mouse TrueBlot	88-8887-31	Rockland Antibodies	RRID:AB_2614895	1:1000 (WB)
Rabbit TrueBlot	88-8886-31	Rockland Antibodies	RRID:AB_2614893	1:1000 (WB)
Goat anti-rabbit	Ab205718	Abcam	RRID:AB_2819160	1:1000 (dot blot)

### Brain tissue co-immunoprecipitation

Manual IP was done using a mass spectrometry-compatible magnetic immunoprecipitation IP kit (#90409, Invitrogen) with beads conjugated to an HT7 total tau antibody (#MN1000, ThermoFisher) according to the manufacturer’s directions; 500 μg of protein from each sample was combined with 5 μg of antibody and diluted to 500 μl with IP-MS Cell Lysis buffer (#90409) before incubating overnight at 4°C with rotary mixing. Protein A/G magnetic beads were washed with IP-MS Cell Lysis buffer in low protein binding microcentrifuge tubes and the immune-complex containing samples were then added and incubated at room temperature for 1 h with constant agitation. The beads were collected using a magnetic stand and the flow-through saved for downstream analysis. Beads were then washed a total of six times for 15 min each with mass spectrometry (MS)-grade water.

### On-bead trypsin digestion and peptide purification

Lyophilized trypsin (#V5111, Promega) was resuspended in Trypsin Resuspension buffer (#V5111, Promega) to a concentration of 0.2 μg/μl. We then added 100 μl of Trypsin Digestion buffer (20 mm Tris HCl pH 8.0, 2 mm CaCl_2_) and 5 μl of trypsin solution to each tube. Bead-containing samples were incubated in a Thermoshaker (Eppendorf) for 4 h at 37°C, shaking at 1200 rpm. The supernatant was then removed and the beads discarded. An additional 1 μg of trypsin was added to each sample followed by an additional digestion overnight for 37°C at 750 rpm on the Thermoshaker. Samples were then acidified to 1% trifluoroacetic acid before purification with OMIX C18 pipette tips (ThermoFisher). OMIX C18 peptide purification was conducted with 5× prewash buffer (80% acetonitrile, 0.1% trifluoroacetic acid, Milli-Q H_2_O to final volume) treatments, 5× wash buffer (0.1% trifluoroacetic acid, Milli-Q H_2_O to final volume) treatments, and 2× elution buffer (60% acetonitrile, 0.1% trifluoroacetic acid, Milli-Q H_2_O to final volume) treatments. Samples were then lyophilized and stored at −80°C until needed.

### Mass spectrometry and data analysis

**Figure 1.**
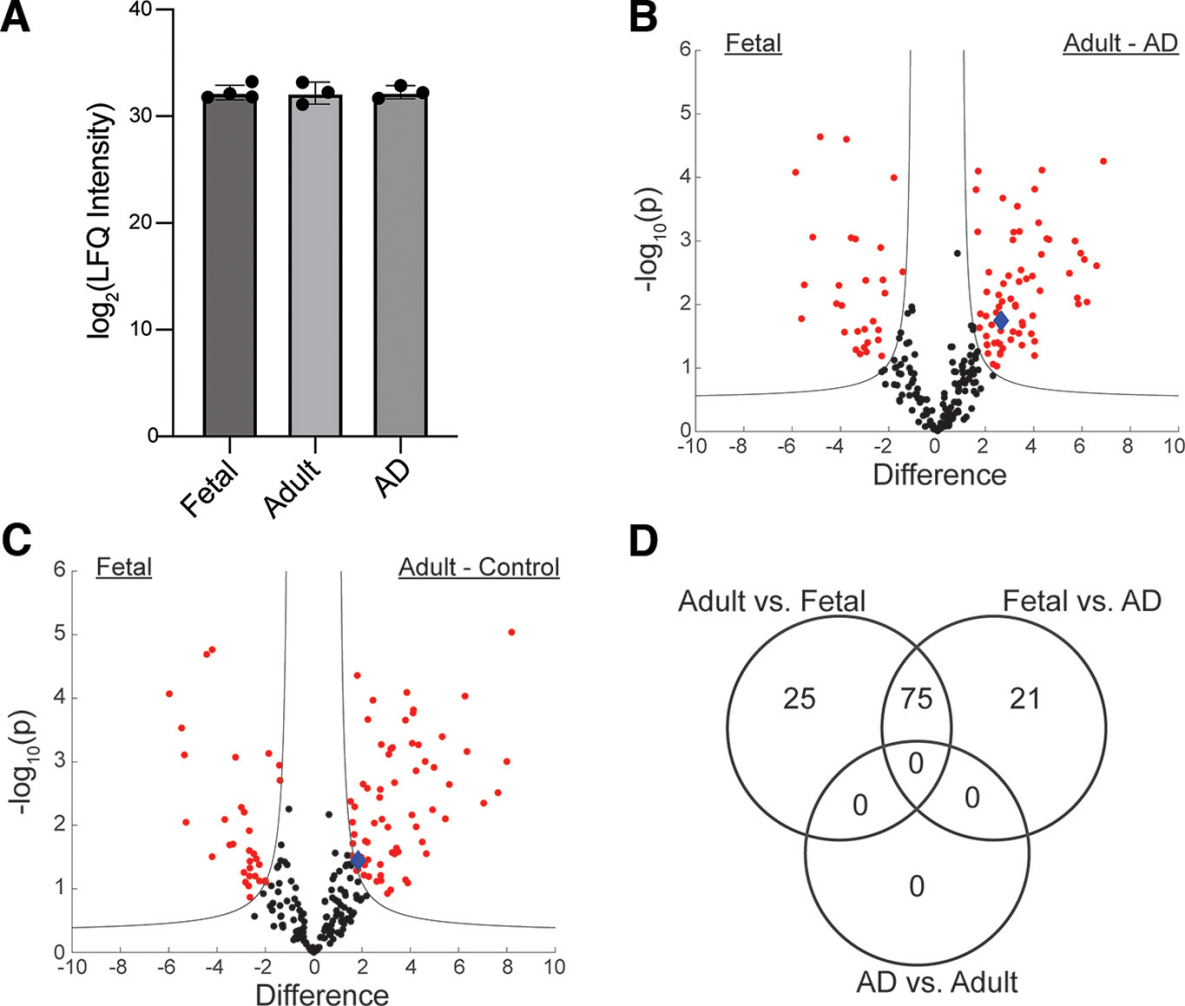
Tau interactome in human fetal, adult, and Alzheimer’s disease brain. ***A***, Total tau levels are not significantly different in immunoprecipitated samples from different groups, *y*-axis shows log_2_ transformed LFQ intensity. Volcano plot showing differentially expressed genes (red) between fetal and AD (***B***), fetal and adult control (***C***), and number of shared genes between comparisons (***D***). Significant interactors are determined using a permutation-based FDR (0.05), 14-3-3-β is indicated as a blue diamond. The horizontal axis in all volcano plots shows the difference of log_2_ transformed LFQ intensity as previously described (Zhang et al., 2019). All differentially expressed genes are shown in Extended Data Table 1-1.

Purified lyophilized peptides were dissolved in 15 μl loading solvent [0.1% TFA in water/CAN (98:2, v/v)] and 4 μl was injected for liquid chromatography tandem mass spectrometry (LC-MS/MS) analysis on an RSLCnano system connected to a QExactive HF mass spectrometer (Thermo). Trapping was done at 10 μl/min for 4 min in loading solvent on a 25 mm C18 trapping column (New Objective). Peptides were then eluted using a nonlinear increase from 2% to 56% solvent B [0.1% formic acid in water/acetonitrile (2:8, v/v)] over 160 min at a constant flow rate of 500 nl/min on a 200 cm column at 37°C. The resulting data were analyzed using the Andromeda search engine in MaxQuant (version 1.6.43.10) with default search settings including false discovery rate (FDR) = 1% at both peptide and protein levels, mass tolerance for precursor ions at 4.5 ppm, and a mass tolerance for fragment ions at 20 ppm and 0.5 Da. Two missed cleavages were allowed. Carbamidomethylation of cysteine residues was set as a fixed modification. Oxidation of methionine and N-terminal acetylation were set as variable modifications. Only proteins with at least one unique or razor peptide were retained in both shotgun searches. We used Perseus (version 1.6.14.0) for downstream analysis including removal of reverse database hits, potential contaminants and IDs identified only by sites. Intensities were log_2_ transformed and normalized for each sample by subtracting the median label free quantification (LFQ) intensity as previously described (Zhang et al., 2019). Replicate samples were grouped, and sites with less than three valid values in at least one group removed. Missing values were imputed from a normal distribution around the detection limit. Differential analysis was done using the Perseus package with FDR = 0.05 and S_0_ = 1 based on published guidelines (Zhang et al., 2019). Because of the large number of statistical tests involved in this analysis, exact statistics for each protein are shown in Figure 1, summarized in Table 3, and listed in Extended Data Table 1-1.

**Table 3:** Statistical table

	Data structure	Test used	Location	Power
a	Normal distribution	Permutation-based FDR	Extended Data Table 1-1	80% for 2-fold difference, α = 0.043
b	List	FDR enrichment	Extended Data Table 4-1	NA
c	List	FDR enrichment	Extended Data Table 4-2	NA
d	Normal distribution	*t* test	Figure 2*B*	90% power for 2-fold difference, α = 0.001

10.1523/ENEURO.0503-22.2023.tab1-1 Extended Data Table 1-1 List of all proteins and statistical analyses (shown in Fig. 1A–C). Download Table 1-1, DOC file.

### Gene ontology and protein domain analysis

Gene ontology (GO) analysis (biological process complete, molecular function complete, and cellular component complete) was conducted using the Panther database’s enrichment overrepresentation test (Fisher’s exact and Bonferroni correction for multiple testing; Mi et al., 2019; Thomas et al., 2022). Generation of protein networks and the protein domain enrichment analysis was conducted using STRING-db using high confidence (0.900) and hiding disconnected nodes from the final networks (Szklarczyk et al., 2015).

### Analysis of known 14-3-3 interactomes

Known 14-3-3 interactomes were obtained from BioGRIDv4.

### HEK 293T transfection plasmids

Plasmids for 0N3R tau and 0N4R tau were the kind gift of Gloria Lee. A pcDNA3 FLAG HA 14-3-3 β plasmid was a gift from William Sellers (Addgene plasmid #8999; http://n2t.net/addgene:8999; RRID:Addgene_8999). All plasmids were verified by Sanger sequencing and/or long-read sequencing. Plasmids were grown using ByLss cell transformation procedures and extracted using the EndoFree Plasmid Maxi kit (QIAGEN, 12362). Final nucleic acid concentrations were determined using a NanoDrop (Thermo, ND-2000) after washing using QIAGEN tips, air drying overnight, and resuspending in endotoxin-free buffer before storing at −20°C.

### Cell culture, transfection, and lysis

HEK 293T (CRL-1573) cells were obtained from ATCC. Cells were cultured in Eagle’s minimum essential medium [EMEM (ATCC, 30-2003)] with 10% heat-inactivated fetal bovine serum (Invitrogen, F4135). Cells were passaged every 3–5 d using Trypsin-EDTA (Invitrogen, 25200056). All experiments were done with cells at 10 passages or earlier with regular testing for mycoplasma. Cell transfection was done as described previously (Takahashi, 2015) using a CaCl_2_/HeBS buffer with 5-μg total expression plasmid per 100-mm dish. At the completion of the transfection protocol, cells were harvested and flash frozen as described previously (Litovchick, 2019) to preserve posttranslational modifications and protein-protein interactions before storing at −80°C. No cell treatments were necessary to ensure generation of phosphorylated tau, as HEK293 cells have been shown to extensively phosphorylate tau to the point of spurring aggregation, likely through endogenous GSK-3β activity (Houck et al., 2016). Cell lysates were prepared as described previously (Takahashi, 2015) using modified lysis buffer (137 mm NaCl, 20 mm Tris–HCl, pH 8.0, 10% glycerol, 1% Triton X-100) with DNase I (Millipore Sigma, DN25-10MG) and HALT (Thermo, 78446) protease and phosphatase inhibitor followed by centrifugation at 15,000 × *g* for 30 min at 4°C.

### Co-immunoprecipitation and Western blotting

We performed co-immunoprecipitation from cell lysates per manufacturer guidelines using Pierce Protein A/G magnetic meads (Thermo, #88802) and the anti-FLAG M2 antibody (MilliporeSigma, F1804). We set up our IP reactions as follows: 500-μg cell lysate, 50 μl (1.5 mg) magnetic beads, and 10 μg anti-FLAG M2 in a total reaction size of 500 μl. We eluted by incubating with a glycine-based elution buffer (pH 2.6, 0.2 m glycine•HCl) at room temperature before neutralizing with 5 m NaOH. Gel electrophoresis and membrane transfer for Western blotting was accomplished using the Mini-PROTEAN Tetra Cell system (Bio-Rad, 1660828EDU) with 2× Laemmli sample buffer and 2-Mercaptoethanol [BME (Bio-Rad, 1610710XTU)]. Proteins were transferred onto Immun-Blot PVDF membrane (Bio-Rad, 1620177) and incubated for 10 min in EveryBlot blocking buffer (Bio-Rad, #12010020) before proceeding to primary and horseradish peroxidase (HRP) secondary antibody staining in the respective blocking buffers. We performed primary antibody incubations overnight at 4°C with gentle rocking, and secondary antibody incubations at room temperature for 1 h with gentle rocking. We used HT7 (Thermo, #MN1000) to detect tau and anti-14-3-3 β (Abcam, ab15260) to detect 14-3-3 β. The latter antibody enabled us to verify that the anti-FLAG pulldown was successfully immunprecipitating 14-3-3-β. We used TrueBlot HRP secondary antibodies (Rockland Antibodies, 88-8887-31/88-8886-31) to detect the protein of interest when interference with heavy and light antibody chains or protein A/G contamination was a concern. Imaging was conducted on the Bio-Rad ChemiDoc Imager.

### Production of ^15^N-labeled Tau441

Human Tau441 was produced in *Escherichia coli* BL21 (DE3) cells (Novagen) using the pET29b vector (a gift from Peter Klein, Addgene plasmid #16316, RRID:Addgene_16316; Hedgepeth et al., 1997). Cells were grown at 37°C and 180 rpm in M9 minimal medium containing 4 g/l glucose, 1 g/l [^15^N] ammonium chloride, 1 mm MgSO_4_, 0.1 mm CaCl_2_, MEM vitamin cocktail (Sigma), and kanamycin (100 μg/ml). Nonlabeled tau441 was produced using the same methods, but without ^15^N-containing growth medium. The induction was delayed until the optical density (OD_600_) reached 1.2 and initialized by addition of 0.4 mm of isopropyl β-D-1-thiogalactopyranoside (IPTG) and continued at 37°C for 3 h. Cells were harvested by centrifugation (5000 × *g*, 20 min, 4°C) and stored at −80°C. The harvested cells were resuspended in buffer (50 mm NaPO_4_, 2 mm EDTA and 2 mm DTT, pH 6.8), and supplemented with protease inhibitor cocktail (Complete, Roche), lysozyme and DNase I. The resuspended cells were disrupted by sonication, and the cell debris was separated by centrifugation (80,000 × *g*, 45 min, 4°C). The supernatant was incubated at 75°C for 15 min, and the soluble proteins were isolated by centrifugation (80,000 × *g*, 45 min, 4°C) and purified by cation exchange chromatography (HiTrap SP, GE Healthcare) with a gradient of 0–1 m NaCl. The eluted fractions containing Tau441 were pooled and further purified by size exclusion chromatography (HiLoad 16/600 Superdex 200 pg, GE Healthcare) in buffer (50 mm NaPO_4_, 2 mm EDTA and 2 mm DTT, pH 6.8). The purified protein was analyzed using SDS-PAGE and concentrated using Amicon Ultra-10K centrifugal filters.

### *In vitro* tau phosphorylation

We used two different *in vitro* phosphorylation techniques because of inherent differences between nuclear magnetic resonance (NMR) and mass photometry and large variations in scalability of available kinase systems. For large-scale *in vitro* phosphorylation of ^15^N-labeled tau441 (^15^N-4R tau) bound for analysis by NMR, we used a two-stage process by which 3 μg of activate MEK1 (MilliporeSigma, #14-429) was used to activate 50 μg of ERK2 (MilliporeSigma, #14-536) before incubation with 4R tau. MEK1 was activated overnight at 30°C with constant agitation in phosphorylation buffer (200 μl; 50 mm HEPES•KOH, pH 8.0, 12.5 mm MgCl_2_, 50 mm NaCl, 1 mm DTT, 1 mm EGTA) with ATP (2.5 mm) and HALT Protease and Phosphatase Inhibitor Cocktail (ThermoScientific, #78440) added just before use. We then incubated 2 mg ^15^N-4R tau with 50 μg activated ERK2 for 4 h at 37°C with constant agitation (ATP was added to maintain concentration at 2.5 mm despite increase in volume). We heated the sample at 75°C for 15 min to inactivate the kinases before centrifugation at 20,000 × *g* for 15 min, allowing the collection of clarified phosphorylated tau in the supernatant. Following Danis and colleagues, we confirmed phosphorylation efficiency and integrity of ERK2-phosphorylated tau with a 12% SDS-PAGE before storing at −8°C (Danis et al., 2016). We chose ERK2 for this application because of the impracticability of phosphorylating sufficient amounts of ^15^N tau for NMR using PKA and because, based on previous NMR studies, ERK2 phosphorylation accurately represents the tau phosphorylation state in the AD brain (Qi et al., 2016). The non-^15^N 4R tau used for small-scale chemical *in vitro* crosslinking was phosphorylated using cAMP-dependent protein kinase (PKA, catalytic subunit, New England Biolabs, #P6000S) according to manufacturer guidelines (25uL reaction at 30°C for 2 h with 200 μm ATP) but substituting the provided Tris-containing “NEBuffer” with an amine-free version (50 mm HEPES, 10 mm MgCl_2_, 2 mm DTT, pH 7.5) to allow for unhindered downstream chemical crosslinking. We heated the samples to 65°C for 20 min followed by centrifugation at 20,000 × *g* for 15 min to inactivate and clear PKA from our sample. We confirmed phosphorylation of tau by PKA using nitrocellulose dot blots with both a total phospho-(Ser/Thr) antibody (Cell Signaling, #9631) and a phospho-tau Ser214 antibody (Abcam, ab170892).

### *In vitro* chemical crosslinking using BS^3^

We used the noncleavable chemical crosslinker BS3 (bis(disuccinimidyl) suberate; Thermo, #21586) according to manufacturer guidelines to perform *in vitro* amine-amine crosslinking of purified 4R tau with purified recombinant 14-3-3 β (LSBio, LS-G96740-100). Crosslinking was performed at 20-fold molar excess (5 mg/μl protein concentration, 100-μl reaction size) in conjugation buffer (1× PBS, pH 8.0) for 30 min at room temperature with gentle agitation before quenching with 1 m Tris•HCl (pH 7.5) to a final concentration of 2 mm with 15 min of incubation at room temperature. Samples not used immediately were stored at −80°C.

### Mass photometry

Mass photometry (MP) experiments were performed on a Refeyn TwoMP mass photometer (Refeyn Ltd). Microscope coverslips (24 × 50 mm, Thorlabs Inc.) were cleaned by serial rinsing with Milli-Q water and HPLC-grade isopropanol (Sigma-Aldrich) followed by drying with a filtered air stream. Silicon gaskets (Grace Bio-Labs) to hold the sample drops were cleaned in the same procedure immediately before measurement. All MP measurements were performed at room temperature using Dulbecco’s PBS (DPBS) without calcium and magnesium (ThermoFisher). The instrument was calibrated using a protein standard mixture: β-amylase (Sigma-Aldrich, 56, 112, and 224 kDa), and thyroglobulin (Sigma-Aldrich, 670 kDa). Before each measurement, 15 μl of DPBS buffer was placed in the well to find focus. The focus position was searched and locked using the default droplet-dilution autofocus function after which 5 μl of protein was added and pipetted up and down to briefly mix before movie acquisition was promptly started. Movies were acquired for 60 s (6000 frames) using AcquireMP (version 2.3.0; Refeyn Ltd) using standard settings. All movies were processed and analyzed using DiscoverMP (version 2.3.0; Refeyn Ltd).

### Nuclear magnetic resonance

^15^N-labeled Tau-441 proteins were exchanged into NMR buffer (2 mm DTT, 2 mm EDTA, 50 mm NaPO_4_, pH 6.8, 10% D_2_O) and concentrated to 100 μm. ^15^N/^1^H HSQC spectra were acquired at 293 K on a Bruker AVANCE NEO 600 MHz NMR spectrometer with a gradient cryoprobe, processed using NMRPipe (Delaglio et al., 1995), and analyzed using POKY (Lee et al., 2021). Previously reported chemical shift assignments for ^15^N-Tau-441 (BMRB ID: 50701; Landrieu et al., 2006; Narayanan et al., 2010) were used to assign isolated correlation peaks. Spectra were collected for Erk2-phosphorylated ^15^N-Tau-441 alone and after addition of 14-3-3β In NMR buffer at a Tau:14-3-3β molar ratio of 1:2. Peak intensities were scaled for determination of intensity ratios.

### Computational prediction of 14-3-3 binding sites

The human Tau441 (2N4R) sequence was downloaded from Uniprot and then entered into the publicly available 14-3-3-pred algorithm via web interface. Only high-confidence sites are reported (identified by all three algorithms used by the program).

### Protein alignment and comparison of interactome

Alignment of 14-3-3 isoforms was done using the Clustal-O algorithm in Uniprot. The human interactome (BioGRID H. sapiens v.4.4) was downloaded from the BioGRID server and the percentage of shared interactions was calculated pairwise using an Excel spreadsheet.

### Statistics

Analysis of mass spectrometry data was done using permutation based-FDR and term-enrichment analysis according to established algorithms described in the Mass spectrometry and data analysis above. Mass photometry data were analyzed using Student’s *t* test in GraphPad Prism. A statistical table is provided in Table 3.

## Results

### Co-immunoprecipitation-mass spectrometry

We first used co-immunoprecipitation with mass spectrometry in postmortem human fetal, adult and Alzheimer’s disease brain to identify differences in the tau interactomes between these conditions. We used bead-linked anti-total tau antibodies to immunoprecipitate tau from ten frozen samples of human fronto-parietal cortex (four fetal controls, three adult controls, three Alzheimer’s disease). Each sample is a distinct piece of brain tissue from a different individual (10 individual distinct donors). The purpose of this study was to directly compare tau in fetal and AD brain tissue, so we sought to maximize the number of fetal brains to increase the power of the comparison. As a result, the study is deliberately underpowered for a direct comparison between adult control and Alzheimer’s disease brain. A total of 1093 proteins were detected, of which 234 were quantifiable. As expected, the levels of tau in each immunoprecipitated sample did not differ significantly (Fig. 1*A*). Of these, 96 differed between fetal and Alzheimer’s disease (Fig. 1*B*), and 100 between fetal and adult control (Fig. 1*C*). Interestingly, we observed no significant differences in the tau interactome between adult and AD brain tissue. There were 75 proteins shared between the fetal-AD and fetal-adult comparisons, and 46 unique to one or the other comparison (Fig. 1*D*). A complete list of all proteins with individual *p*-values is in Extended Data Table 1-1. We acknowledge that the small sample size of this portion of the study limits statistical power, sample size in this study was driven by the largest number of samples that could practically be run at one time through our co-IP protocol, and by available running times in our mass spectrometry facility, since each technical replicate required a 160-min run time, for a total run time of 80 h (3.3 d).

### Gene ontology enrichment analysis

Since both fetal and AD brain have similar levels of tau phosphorylation, we focused on the divergence of enriched biological processes seen in the fetal and Alzheimer’s disease tau interactome (Yu et al., 2009; Hefti et al., 2019). We used PantherDB to identify enriched gene ontology terms within our differentially interacting protein sets. Proteins increased in the fetal co-immunoprecipitation experiments were enriched for axon extension (GO:45773), consistent with the known role of tau as a microtubule binding protein. Interestingly, we also found enrichments for RNA binding (GO:00003723) and various nucleus-associated cellular components such as paraspeckles (GO:42832), nuclear matrix (GO:16363), and the nuclear speck (GO:16607). In the AD brain, the top enriched terms were neurofilament bundle assembly (GO:0033693), postsynaptic intermediate filament cytoskeleton (GO:0099160), and fructose-bisphosphate aldolase activity (GO:0004332), in the biological process, cellular component and molecular function ontologies, respectively. The top five gene ontology (GO) terms by fold enrichment (FE) are shown in Table 4, with exact *p*-values listed, and the remainder are shown in Extended Data Tables 4-1 and 4-2.

**Table 4:** List of top five enriched gene ontology terms in genes showing increased tau interaction in fetal brain (top) or AD brain (bottom)

	GO	Term	Total	Expected	FE	FDR
Fetal	BP	Positive regulation of axon extension (GO:0045773)	3	0.05	58.66	4.98E-02
	BP	Positive regulation of response to biotic stimulus (GO:0002833)	5	0.25	20.17	3.75E-02
	BP	Negative regulation of biological process (GO:0048519)	19	6.97	2.73	2.51E-02
	MF	Structural molecule activity (GO:0005198)	9	1.04	8.62	1.25E-03
	MF	Protein-containing complex binding (GO:0044877)	10	1.72	5.81	5.80E-03
	MF	RNA binding (GO:0003723)	12	2.18	5.49	2.21E-03
	CC	Paraspeckles (GO:0042382)	3	0.01	>100	2.24E-04
	CC	Nuclear matrix (GO:0016363)	6	0.17	36.31	3.33E-05
	CC	Growth cone (GO:0030426)	5	0.22	22.56	1.43E-03
	CC	Cortical cytoskeleton (GO:0030863)	3	0.15	20.61	4.47E-02
	CC	Nuclear speck (GO:0016607)	6	0.55	10.92	3.88E-03
Alzheimer’s disease	BP	Neurofilament bundle assembly (GO:0033693)	3	0.01	>100	3.28E-04
	BP	Axon target recognition (GO:0007412)	2	0.01	>100	1.38E-02
	BP	Postsynaptic intermediate filament cytoskeleton organization (GO:0099185)	2	0.01	>100	1.37E-02
	BP	Isocitrate metabolic process (GO:0006102)	2	0.02	>100	3.10E-02
	BP	Fructose 1,6-bisphosphate metabolic process (GO:0030388)	3	0.03	>100	1.78E-03
	CC	Postsynaptic intermediate filament cytoskeleton (GO:0099160)	3	0.01	>100	6.80E-05
	CC	Laminin-11 complex (GO:0043260)	2	0.01	>100	3.51E-03
	CC	Neurofibrillary tangle (GO:0097418)	3	0.02	>100	9.71E-05
	CC	Internode region of axon (GO:0033269)	2	0.01	>100	4.70E-03
	CC	Calcium- and calmodulin-dependent protein kinase complex (GO:0005954)	2	0.02	>100	6.11E-03
	MF	Fructose-bisphosphate aldolase activity (GO:0004332)	2	0.01	>100	1.35E-02
	MF	Protein kinase C inhibitor activity (GO:0008426)	2	0.01	>100	1.31E-02
	MF	Structural constituent of postsynaptic intermediate filament cytoskeleton (GO:0099184)	2	0.01	>100	1.28E-02
	MF	Cytoskeletal protein-membrane anchor activity (GO:0106006)	2	0.01	>100	1.74E-02
	MF	Low-density lipoprotein particle receptor binding (GO:0050750)	3	0.08	36.88	1.36E-02

See row b in statistical table for analysis and Extended Data Tables 4-1 and 4-2 for complete lists of all enriched terms.

10.1523/ENEURO.0503-22.2023.tab4-1 Extended Data Table 4-1 Table of all gene ontology term enrichments (AD). Download Table 4-1, DOC file.

10.1523/ENEURO.0503-22.2023.tab4-2 Extended Data Table 4-2 Table of all gene ontology term enrichments (Fetal). Download Table 4-2, DOC file.

### Protein domain analysis

We used STRING to calculate enrichment of specific InterPro or PFAM protein domains in all tau interactors that could be quantified in our experiment. The top enriched InterPro domains were NOPS, fibrinogen α/β/γ chain coiled coil, microtubule associated binding protein tubulin binding, and 14-3-3 domains. Repeating the analysis using PFAM domains produced an identical result. Detailed results, including *p*-values are shown in Table 5. We chose to focus on 14-3-3 family proteins for further analysis given their high level of expression in brain, known role in phospho-protein homeostasis and role in tau interaction in adult brain, and lack of developmental changes in expression (Steinacker et al., 2011; Carlyle et al., 2017; Cornell and Toyo-Oka, 2017; Drummond et al., 2020).

**Table 5:** List of enriched domains

ID	Description	Number	Strength	FDR
PF00076	RNA recognition motif (also known as RRM, RBD, or RNP domain)	17	0.87	1.73E-06
PF00244	14-3-3 protein	6	1.87	5.59E-06
PF04732	Intermediate filament head (DNA binding) region	5	1.79	0.00015
PF00038	Intermediate filament protein	9	1.04	0.0003
PF00538	Linker histone h1 and h5 family	5	1.6	0.00049
PF01391	Collagen triple helix repeat (20 copies)	8	0.96	0.0029
PF00052	Laminin B (Domain IV)	4	1.64	0.0037
PF01979	Amidohydrolase family	4	1.54	0.0064
PF00418	Tau and MAP protein, tubulin-binding repeat	3	1.94	0.0108
PF08075	NOPS (NUC059) domain	3	1.94	0.0108
PF08702	Fibrinogen α/β chain family	3	1.94	0.0108
PF00053	Laminin EGF domain	5	1.15	0.0141
PF08332	Calcium/calmodulin dependent protein kinase II association domain	3	1.81	0.0141
PF13474	SnoaL-like domain	3	1.81	0.0141
PF14534	Domain of unknown function (DUF4440)	3	1.81	0.0141
PF00055	Laminin N terminal (Domain VI)	4	1.34	0.0147
PF13671	AAA domain	4	1.34	0.0147
PF00932	Lamin tail domain	3	1.72	0.0149
PF03953	Tubulin C-terminal domain	4	1.18	0.0422
IPR000504	RNA recognition motif domain	18	0.84	2.95E-06
IPR012677	Nucleotide-binding α-β plait domain superfamily	18	0.81	3.61E-06
IPR035979	RNA-binding domain superfamily	18	0.81	3.61E-06
IPR000308	14-3-3 protein	6	1.87	5.49E-06
IPR023409	14-3-3 protein, conserved site	6	1.87	5.49E-06
IPR023410	14-3-3 domain	6	1.87	5.49E-06
IPR036815	14-3-3 domain superfamily	6	1.87	5.49E-06
IPR018039	Intermediate filament protein, conserved site	9	1.1	9.24E-05
IPR006821	Intermediate filament head, DNA-binding domain	5	1.79	0.0001
IPR039008	Intermediate filament, rod domain	9	1.03	0.00026
IPR005818	Linker histone H1/H5, domain H15	5	1.52	0.00085
IPR011778	Hydantoinase/dihydropyrimidinase	4	1.76	0.0018
IPR008160	Collagen triple helix repeat	8	0.96	0.0029
IPR000034	Laminin IV	4	1.64	0.0036
IPR005819	Linker histone H1/H5	4	1.64	0.0036
IPR011059	Metal-dependent hydrolase, composite domain superfamily	4	1.54	0.0063
IPR043129	ATPase, nucleotide binding domain	7	0.97	0.007
IPR006680	Amidohydrolase-related	4	1.5	0.0076
IPR001084	Microtubule associated protein, tubulin-binding repeat	3	1.94	0.0101
IPR012290	Fibrinogen, α/β/γ chain, coiled coil domain	3	1.94	0.0101
IPR012975	NOPS	3	1.94	0.0101
IPR013543	Calcium/calmodulin-dependent protein kinase II, association-domain	3	1.81	0.0151
IPR008211	Laminin, N-terminal	4	1.34	0.0201
IPR001322	Lamin tail domain	3	1.72	0.022
IPR036415	Lamin tail domain superfamily	3	1.72	0.022
IPR002049	Laminin EGF domain	5	1.07	0.0286
IPR037103	Tubulin/FtsZ, C-terminal domain superfamily	4	1.22	0.0428

### *In vitro* co-immunoprecipitation and Western blotting

**Figure 2.**
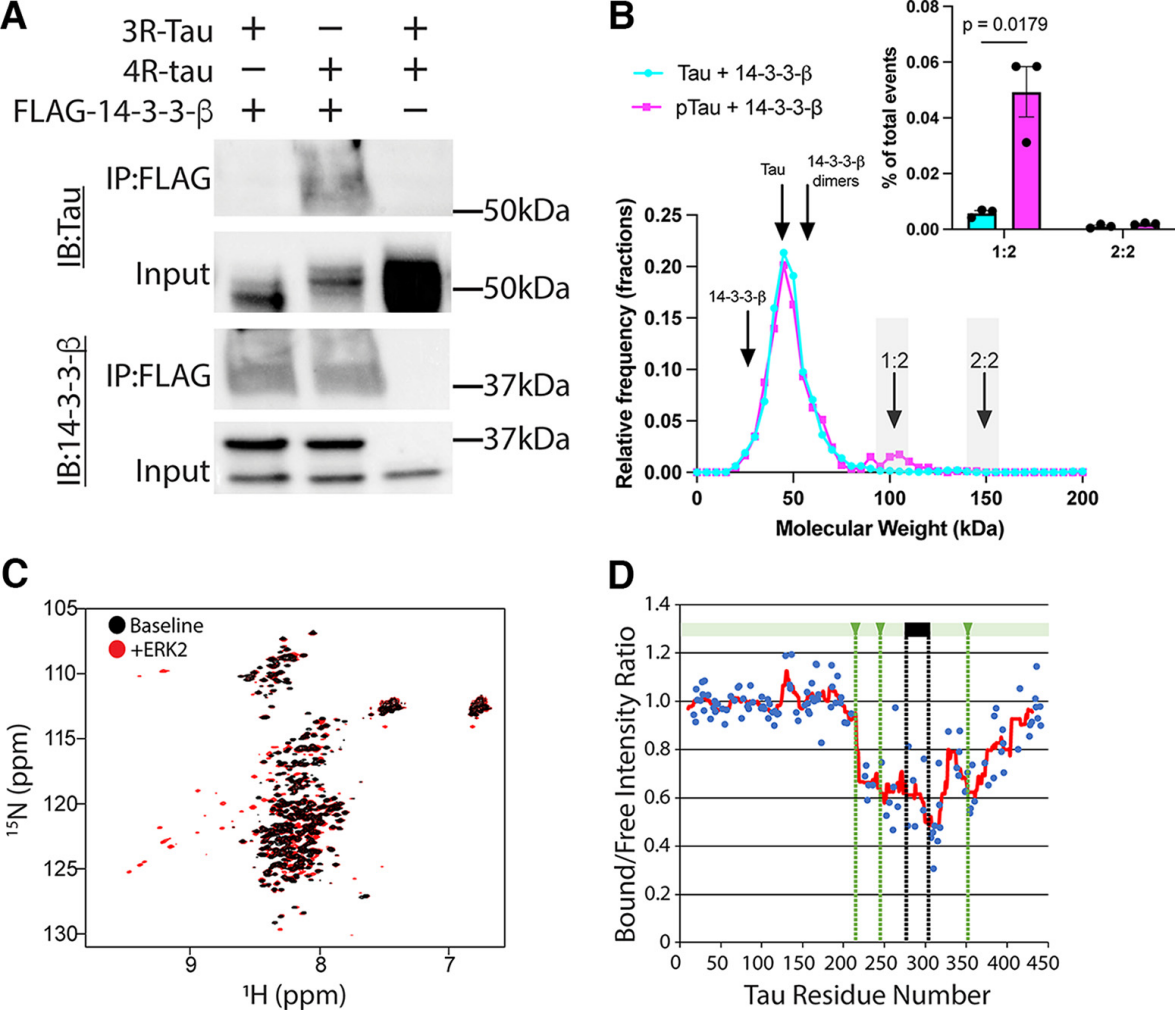
Tau-14-3-3-β interaction depends on tau splicing and phosphorylation. ***A***, HEK 293T cells were transfected with the indicated constructs, immunoprecipitated with anti-FLAG antibodies, and then probed with the indicated antibodies. Uncropped blots are shown in Extended Data Figure 2-1, and an additional replicate (uncropped) in Extended Data Figure 2-2. ***B***, Representative mass photometry density plots of phosphorylated (magenta) and unphosphorylated (cyan) tau 14-3-3-β one representative plot is shown out of three replicates, gray bars indicate windows of predicted MW ±10 kDa and quantification across three replicates is shown in upper right using Student’s *t* test, validation of phosphorylation is shown in Extended Data Figure 2-3 and a phosphorylated tau only plot on the same axes is shown in Extended Data Figure 2-4. ***C***, Recombinant 15N-labeled tau441 NMR with and without ERK1 phosphorylation (validated in Extended Data Fig. 2-5). ***D***, Phosphorylated 15N-labeled tau 441 showing residue shifts after incubation with 2:1 ratio of 14-3-3-β to tau, green lines indicate predicted 14-3-3-pred binding sites, black indicates variably spliced second microtubule binding repeat (R2).

We then sought to validate the interaction between tau and 14-3-3 proteins, focusing on the protein with the greatest difference between fetal and AD brain (14-3-3-β, shown as blue diamonds in Fig. 1*A–C*). We first transfected HEK 293T cells with either 3R or 4R tau plasmids and co-transfected with FLAG-14-3-3-β. These specific isoforms were selected because, based on existing data with other 14-3-3 isoforms, 14-3-3 protein binding regions on tau are thought to flank the variably spliced second microtubule binding repeat (Neves et al., 2020). We were able to co-immunoprecipitate 4R but not 3R tau with 14-3-3 β (Fig. 2*A*). In addition to the plasmid sequencing studies (see Materials and Methods) the Western blotting provides additional verification of the presence of different tau isoforms based on the difference in migration between 3R and 4R tau (Fig. 2*A*). Uncropped blots are shown in Extended Data Figure 2-1 and an additional replicate in Extended Data Figure 2-2.

10.1523/ENEURO.0503-22.2023.f2-1 Extended Data Figure 2-1 Uncropped western blot images for Figure 2*A*. Red boxes indicate irrelevant bands from unrelated experiment. Boxes indicate bands shown in Figure 2*A*. Additional bands in top left, and bottom left, right side represent tau degradation products. Download Figure 2-1, TIF file.

10.1523/ENEURO.0503-22.2023.f2-2 Extended Data Figure 2-2 Replication of co-IP in Figure 2*A*, with uncropped blots. Download Figure 2-2, TIF file.

**Figure 3.**
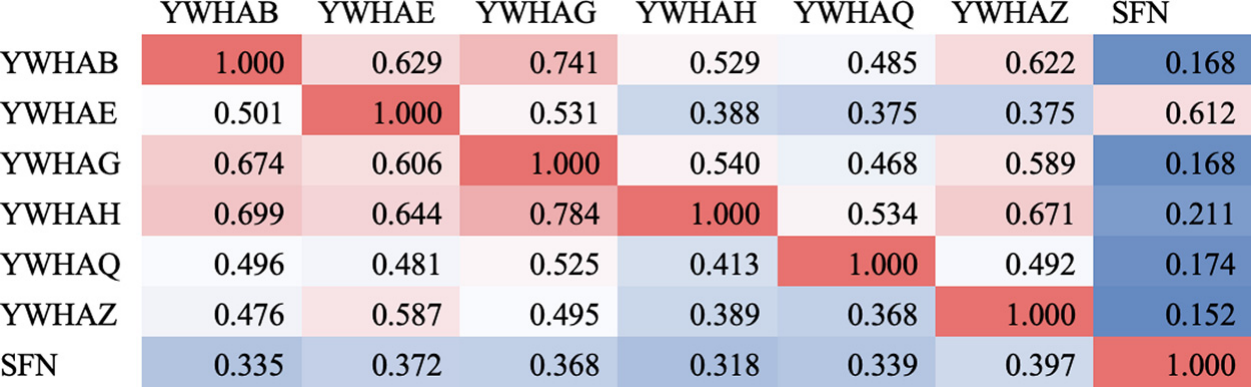
Fraction of shared interactors between human 14-3-3 isoforms (BioGRIDv4.4).

10.1523/ENEURO.0503-22.2023.f2-3 Extended Data Figure 2-3 Validation of tau phosphorylation by PKA for mass photometry. We added 1 μg of the phosphorylated, recombinant tau to a nitrocellulose membrane and probed with anti-tau Ser214 and total pSer/pThr antibodies as indicated. Control, unphosphorylated tau. Download Figure 2-3, TIF file.

10.1523/ENEURO.0503-22.2023.f2-4 Extended Data Figure 2-4 Mass photometry histogram of ptau only, as in Figure 2*B*. Download Figure 2-4, TIF file.

10.1523/ENEURO.0503-22.2023.f2-5 Extended Data Figure 2-5 Validation of tau phosphorylation by ERK2 for NMR. We loaded 10 μg of phosphorylated, recombinant tau and stained the resulting gel with Coomassie blue as an initial validation of phosphorylation efficiency (see Fig. 2*C* and text for site-specific mapping of phosphoepitopes). Control, unphosphorylated tau. Download Figure 2-5, TIF file.

### Mass photometry

To further characterize this interaction, we performed *in vitro* chemical crosslinking on purified 4R (tau441) tau with and without PKA phosphorylation and 14-3-3-β and used mass photometry to measure the distribution of molecular mass in the resulting mixture. Before sample preparation, we confirmed phosphorylation efficiency by dot blotting and probing with total pSer/Thr and tau Ser214 antibodies (Extended Data Fig. 2-3). We observed a protein complex at the molecular weight predicted for 1:2 tau:14-3-3-β stoichiometry, but not at that predicted for 2:2 stoichiometry, and this complex appeared only when phosphorylated tau was co-incubated with 14-3-3-β, but not with unphosphorylated tau with 14-3-3-β (Fig. 2*B*) or phosphorylated tau alone (Extended Data Fig. 2-4). Quantification of this peak across three replicates (Fig. 2*B*, upper right) showed a statistically significant difference between phosphorylated and unphosphorylated tau for the 1:2 stoichiometry (*p* = 0.018), but not 2:2.

### Computational prediction of 14-3-3 binding sites on the tau protein

We then used 14-3-3pred to identify putative 14-3-3 protein binding sites on the tau protein. Using the full length (2N4R, Tau441) sequence, we identified three high-confidence binding sites, which include Ser214, Ser245, and Ser352, respectively, these are indicated with green lines on Figure 2*D*.

### Nuclear magnetic resonance

We then used nuclear magnetic resonance (NMR) to map 14-3-3-β interaction sites on the tau protein. Given the extremely low throughput of NMR experiments, we decided to focus on the interaction of 2N4R tau with 14-3-3-β. Since 2N4R tau is the longest isoform present in the human central nervous system, this enabled us to identify any potential interaction sites, including those within variably spliced regions of the tau protein. Phosphorylation of tau using activated ERK2 produced the expected peak shift pattern for phosphorylated amino acid residues pT50, pT69, pT153, pT175, pT181, pS191, pS199, pT205, pT231, pS235, pS396, pS404, and pS422 (Fig. 2*C*; Extended Data Fig. 2-5). When we co-incubated phosphorylated tau with 14-3-3-β, we found a decrease in intensity across the proline-rich and microtubule binding domains of tau (Fig. 2*D*). This includes the three high-confidence binding sites predicted by 14-3-3-pred (Ser214, Ser245, and Ser352), shown as green vertical dashed lines in Figure 2*D*. As expected, these sites span the second microtubule binding repeat, which is present in 4R tau, which interacts with 14-3-3-β, but not in 3R tau, which does not (Fig. 2*D*, black box and black dashed vertical lines; Madeira et al., 2015; Arendt et al., 2016).

### Comparison of 14-3-3 isoform interactomes

Since the 14-3-3 family proteins show a high degree of sequence homology, we sought to determine the plausibility of our result showing that the ability of some isoforms but not others differs between fetal and AD brain. We therefore compared the known interactomes of the 14-3-3 family proteins using data from BioGRIDv4. The fraction of shared interactors ranges from 0.152 to a maximum of 0.699 (Fig. 3).

## Discussion

Our data show that the greatest differences in the tau interactome are between fetal and adult brain tissue, either control or Alzheimer’s disease, rather than between Alzheimer’s disease and adult control brain. Although, as noted below, the certainty of these conclusions is limited by the necessarily small sample size in the mass spectrometry portion of this study. In particular, we found that the interaction between phosphorylated tau and 14-3-3 family proteins differs markedly between fetal and adult brain, and that this likely depends on the age-dependent alternative splicing of MAPT’s exon 10.

Previous studies of the tau interactome have used genetically modified tagged tau and/or animal models, limiting the physiological relevance of the results (Liu et al., 2016; Tracy et al., 2022). Comparing our list of quantified proteins across all age groups to that reported by Tracy et al., who used APEX-labeled tau in human iPSC-derived neurons, we find that 120 proteins are unique to our study, 57 are shared, and 210 are unique to Tracy et al. (2022). This both supports the validity of our data and underlines the importance of combining observational human tissue-derived data with functional *in vitro* studies using genetically engineered tau constructs. The limited data available in human tissue focus on adult brains, either control or with neurodegenerative tauopathies, and have a limited number of cases (Drummond et al., 2020). None of these studies consider developmental changes or take advantage of the unique resilience of the developing brain to tau toxicity. In addition, modifying tau with either tags or with biotinylation enzymes has the potential to disrupt or change the tau interactome because of steric effects and/or the effect of tau protein overexpression.

Our findings are consistent with previous data suggesting that tau interacts with 14-3-3 family proteins in both human and animal brain (Layfield et al., 1996; Hashiguchi et al., 2000; Umahara et al., 2004; Sugimori et al., 2007; Sadik et al., 2009a,b; Sluchanko et al., 2009b; Sluchanko and Gusev, 2011; Qureshi et al., 2013). Our findings do, however, differ from the existing data in several key ways. First, the specific isoforms we identified as differentially interacting with tau in the AD brain (14-3-3-β, γ, and η) have not been systematically studied as tau-interacting proteins, although they have been identified as tau interacting proteins in human brain (Drummond et al., 2020). 14-3-3-β has been described as a component of human neurofibrillary tangles, but its interaction with tau has not been otherwise studied (Sugimori et al., 2007). The role of 14-3-3-γ and η in tau pathology remain unknown. Interestingly, previous studies of 14-3-3-ζ, which in our dataset interacts equally with fetal and adult tau, shows interaction with phosphorylated 3R tau (Sluchanko et al., 2009a,b). Although the seven 14-3-3 family proteins in humans show a high degree of structural homology, their known interactomes vary significantly (Fig. 3). Another potential reason for differences between our findings and those reported in tau interactions with other 14-3-3 family members is that we used a proline directed kinase (ERK2) to phosphorylate tau for our NMR studies, as opposed to the more commonly used PKA. Based on published data, ERK2 drives a tau phosphorylation profile representative of the human brain while also allowing for significant reaction upscaling, which is critical for techniques such as NMR (Danis et al., 2016; Qi et al., 2016). As a proline directed kinase, ERK2 would poorly phosphorylate serines or threonines in the classic 14-3-3 binding motif pS/pTXP. However, recent data using mass spectrometry to map tau posttranslational modifications in both Alzheimer’s disease and control adult brain suggests that the predicted 14-3-3 binding motifs in “tau pred” (Ser214, Ser245, and Ser352) show low-to-minimal levels of phosphorylation (Wesseling et al., 2020).

Our study has several limitations. First, although we required all our cases to have short postmortem intervals (<24 h), we could not, because of the smaller number of specimens, include this is a covariate in our differential expression analysis for the proteomics studies. It is therefore possible, albeit unlikely, that postmortem interval played a role in some of the observed interactions noted above. Our co-IP-MS protocols are optimized to detect strong and persistent interactions, it should be noted that there are few membrane proteins in our tau interactome, suggesting that this method does not capture more transient interactions with membrane proteins. Although highly tractable and extensively used for biochemical studies, HEK 293T cells do not fully replicate the cellular environments of human neurons, and overexpressing proteins can lead to spurious interactions. The fact that we saw the same interaction with a consistent effect of splicing in both our co-IP-MS and transfection experiments strongly supports the validity of our data.

Our data represent the first systematic characterization of the tau interactome in human fetal, adult, and Alzheimer’s disease brain. We report a unique dataset of the human tau interactome in fetal, adult, and Alzheimer’s disease brain, and provide the first systematic description of tau-14-3-3-β interaction in the human brain. We also present the first description of an isoform dependent tau-14-3-3-β protein interaction.

## Synthesis

Reviewing Editor: Karl Herrup, University of Pittsburgh School of Medicine Decisions are customarily a result of the Reviewing Editor and the peer reviewers coming together and discussing their recommendations until a consensus is reached. When revisions are invited, a fact-based synthesis statement explaining their decision and outlining what is needed to prepare a revision will be listed below. The following reviewer(s) agreed to reveal their identity: Charles White, Amantha Thathiah.

The authors studied the tau interactome in human autopsy brain from fetal, normal adult, and Alzheimer disease (AD) subjects using co-immunoprecipitation and mass spec. The goal was to identify potential mechanisms of resilience to phospho-tau aggregation in fetal brain. Fetal and AD tau are equally hyperphosphorylated, yet fetal tau does not develop the toxic inclusions seen in AD brains. Additionally, different tau isoforms are enriched in fetal (3R) and AD (3R+4R) brains. However, the putative protective mechanisms that prevent the aggregation of hyperphosphorylated fetal tau remain unknown. Differences in proteins interacting with tau were investigated and found to differ between fetal brain and both adult and AD brain, but not between adult control and AD brain. Differences were observed in certain isoforms of proteins enriched for 14-3-3 chaperone protein domains, some of which have been reported to be associated neurofibrillary tangles as seen in AD. Moreover, the interaction between 14-3-3 and tau was primarily associated with 4R tau, which is lacking in fetal brain, providing a possible basis for resilience of fetal brain to toxicity associated with aggregated tau.

This is a generally strong paper. The methods and tissue sources are well described, the results are novel and presented clearly, the tables are informative, the discussion is complete - including awareness of the limitations of the study - and the conclusions appear reasonable. The findings have significance for our understanding of mechanisms of resilience to neurodegeneration due to cytotoxic effects of aggregated phospho-tau. That said, there are some items that need attention and incorporation into the manuscript that would strengthen the authors points.

Major concerns

• The authors must offer proof that their pulldown of tau from the different brain samples was equivalent prior to mass spectrometry analysis of the tau interactome. While they have used well-established magnetic protein A/G beads conjugated to a total tau antibody (H7) in their study, they should provide biochemical (Western blot) data showing the input levels of tau from each brain sample and the amount of bead-bound tau for each sample. This is important given that changes in tau levels may directly impact the detection of putative interactors and abundance of the detected interactors, including 14-3-3 proteins. Alternatively, the authors can report protein levels of tau obtained from mass spectrometry analysis to show whether similar levels of tau were pulled down and eluted prior to analysis of tau interactors.

• The sample size for each group is small for a human study - only 3-4 brains per group (e.g., fetal, adult control, and AD) were analyzed. This greatly limits the statistical power and the ability to extrapolate their findings to a broader cohort. For example, the authors are unable to determine whether age, gender, PMI, etc. are factors in the biological findings. The authors recognize this on line #256: “...the study is deliberately underpowered for a direct comparison between adult control and Alzheimer disease brain.” Yet they go on to argue for a lack of change between adult and AD brains (Fig 1C and D, line #6, #259 #317) and do so with more certainty than may be justified from the data. While additional samples would be desirable, this may be impractical for a revision. Nonetheless, the authors should expand their discussion of how the limiting sample size also limits the interpretation of their results and provide more information on the power analysis of the study and/or rationale for the chosen sample sizes.

• The authors should more clearly establish the rationale for pursuing the interaction of tau and 14-3-3 proteins. They state that they chose the 14-3-3 family of proteins because they are “highly expressed in the brain and related to phospho-protein homeostasis”. However, it remains unclear why they think 14-3-3 proteins may be more strongly involved in tau pathogenesis than the other differentially regulated proteins that had higher significance values in their dataset. Are there changes in levels and/or modification of 14-3-3 proteins in adults compared to fetal brains reported in the literature? If not, authors should consider investigating this in their samples to more definitively establish the proposed mechanism. Further, while the claim is made that the authors are the first to elucidate that 14-3-3 protein isoforms γ and η interact with tau (line #336-337), this ignores the earlier work of Drummond et al. (2020, Brain). This earlier work must be acknowledged.

• There is a disconnect between the in vivo (all isoforms), HEK293 (0N3R and 0N4R), and purified protein (2N4R) tau isoforms used throughout the study. The authors need to discuss each choice and possible confounding variables that the N domains may have on their tau interactome analysis. In this same vein, the study would benefit from proper validation of 3R and 4R plasmids with isoform-specific antibodies. These antibodies could also be used to validate the 4R/3R preference of 14-3-3 proteins in human fetal, adult control, and AD brains. This would greatly increase the strength of the study by translating their isoform-preference findings to the human samples.

• Samples were treated with benzonase prior to IP and mass spectrometry analysis. This is a concern because benzonase disrupts DNA/RNA species which are fundamental for several tau interactions. As such, the authors should discuss the rationale for the use of benzonase and how this might have impacted their findings in terms of biasing which differential interactors they observed.

• From the data provided in Fig. 2, it is unclear why the authors claim they have found a 1:2 Tau-14-3-3 complex and not a tau dimer. Please clarify this. Is this based on the total mass? The authors could repeat the same reactions with tau and 14-3-3 separately, and observe the distribution of masses to match with the weight observed in the combined reaction.

• In panel B, the graph shows that 12% of the mass represents the 115kDa species in the PKA phosphorylated control. However, on the no PKA control the tau/14-3-3 monomer peak is 90% of the mass count, which leaves a small peak at the 115kDa that may correspond to about 10%. The representation of a single replicate/graph leaves it unclear the magnitude and reproducibility of these findings. To claim that the formation of this putative 1:2 complex of 115kDa mass is phosphorylation-dependent, the authors should present at least three replications of these experiments, quantify the mass at the 115kDa value and do a t-test of the mass values +/-PKA.

Minor concerns

Introduction

• The Alzheimer’s Disease acronym “AD” is defined twice in lines #31 and #36

Figures

• Figure 1: Clarify the meaning of “Difference” means in the volcano plot axis.

• Figure 2: There is a smear and not a clear band in the bound fraction of the coIPS in panel A. First, please provide uncropped blots. Second, please address the interference of antibody species given that both the FLAG and tau (H7) antibodies are raised in mouse. Third, the authors need to provide more than one replicate to show the validity of the representative coIP.

The plots in panel “B” have different magnitudes of the Y axis. This may deceive us to notice the second peak at 115kDa more in the +PKA condition. Also, the graphs need to have the X and Y axis labeled.

In 2-1 & 2-2: Please clarify the meaning of “control” (unphosphorylated?) in the figure legend.

• Figure 3: This figure is cited for the first time only in the discussion portion of the manuscript. They should include this citation in the results section.

• The authors have stated they performed ’Computational prediction of 14-3-3 binding sites’. However, they have not provided any visual representation of these predicted sites. The authors should consider adding this information to the main figure in order to enrich the manuscript.

• Tables:

Tables 1-1, 1-2, and 1-3 listed in the statistical analysis but not apparent in the manuscript

Table 3 lacks the necessary “Power” column with the confidence intervals for their statistical tests as specified in the journal guidelines.
