# Supplementary material for: Characterization of the Tau Interactome in Human Brain Reveals Isoform-Dependent Interaction with 14-3-3 Family Proteins
Source: eNeuro. 2023 Mar 21;10(3):ENEURO.0503-22.2023. doi: 10.1523/ENEURO.0503-22.2023 (PMC10035768; doi:10.1523/ENEURO.0503-22.2023)
Supplement: Extended Data Table 1-1 — List of all proteins and statistical analyses (shown in Fig. 1A–C). Download Table 1-1, DOC file. [file enu-eN-NWR-0503-22-s01.doc]

**Table 1-1^a^.** List of all proteins and statistical analyses (shown in Figure 1a-c).

|  |  | **Adult vs. Alzheimer** | | **Adult vs. Fetal** | | **Fetal vs. Alzheimer** | |
| --- | --- | --- | --- | --- | --- | --- | --- |
| **Protein ID** | **Gene** | **-log_10_(p)** | **Difference** | **-log_10_(p)** | **Difference** | **-log_10_(p)** | **Difference** |
| P31946 | YWHAB | 0.42 | -0.81 | 1.44 | 1.84 | 1.74 | 2.65 |
| P62258 | YWHAE | 0.32 | -0.66 | 0.63 | 1.66 | 0.88 | 2.31 |
| Q04917 | YWHAH | 0.53 | -1.59 | 0.31 | 1.00 | 1.38 | 2.60 |
| P61981 | YWHAG | 0.20 | -0.39 | 1.46 | 2.25 | 1.59 | 2.64 |
| P27348 | YWHAQ | 0.10 | -0.22 | 0.76 | -0.66 | 0.27 | -0.44 |
| P63104 | YWHAZ | 0.52 | -1.02 | 0.34 | 0.44 | 0.98 | 1.47 |
| P09543 | CNP | 1.20 | 1.19 | 2.35 | 7.04 | 2.01 | 5.85 |
| P43686 | PSMC4 | 0.38 | 0.76 | 0.25 | -0.42 | 0.71 | -1.18 |
| P10809 | HSPD1 | 0.20 | 0.17 | 1.55 | -2.48 | 1.74 | -2.65 |
| P05387 | RPLP2 | 0.20 | -0.30 | 1.36 | -1.46 | 1.40 | -1.16 |
| Q02878 | RPL6 | 0.27 | 0.38 | 0.35 | -0.60 | 0.82 | -0.99 |
| Q99798 | ACO2 | 0.40 | -1.26 | 0.85 | 1.98 | 1.97 | 3.25 |
| P60709 | ACTB | 0.41 | 0.17 | 0.80 | 0.47 | 0.54 | 0.30 |
| P00568 | AK1 | 0.86 | -2.01 | 0.26 | -0.58 | 1.34 | 1.43 |
| P12814 | ACTN1 | 0.11 | -0.49 | 0.59 | 1.59 | 1.36 | 2.07 |
| P35611 | ADD1 | 1.31 | -1.62 | 0.55 | 0.52 | 2.51 | 2.14 |
| P61163 | ACTR1A | 0.14 | -0.21 | 1.21 | 0.85 | 1.02 | 1.06 |
| P02511 | CRYAB | 0.54 | 0.64 | 3.16 | 6.35 | 3.00 | 5.71 |
| Q16352 | INA | 1.21 | 0.99 | 1.13 | 2.79 | 0.68 | 1.81 |
| P63010 | AP2B1 | 0.36 | -0.87 | 0.00 | 0.00 | 0.63 | 0.87 |
| P00505 | GOT2 | 0.28 | -0.31 | 2.86 | 4.24 | 3.04 | 4.54 |
| P25705 | ATP5A1 | 0.05 | -0.12 | 0.24 | 0.28 | 0.22 | 0.40 |
| P06576 | ATP5B | 0.64 | -1.24 | 0.74 | -1.39 | 0.05 | -0.15 |
| P36542 | ATP5C1 | 0.13 | -0.23 | 2.38 | 1.53 | 1.63 | 1.76 |
| Q01813 | PFKP | 0.34 | -0.38 | 2.57 | 2.76 | 3.02 | 3.14 |
| Q08211 | DHX9 | 0.61 | 1.56 | 3.22 | 3.26 | 0.97 | 1.70 |
| Q9Y2J2 | EPB41L3 | 0.02 | 0.02 | 3.20 | 3.19 | 3.14 | 3.17 |
| P98160 | HSPG2 | 0.10 | 0.36 | 0.80 | 1.02 | 0.24 | 0.66 |
| P21810 | BGN | 0.65 | -1.73 | 0.18 | -0.41 | 0.84 | 1.32 |
| P80723 | BASP1 | 0.54 | 0.58 | 1.20 | -2.42 | 1.61 | -3.00 |
| Q9UQM7 | CAMK2A | 0.18 | -0.21 | 3.82 | 4.13 | 4.12 | 4.34 |
| Q13554 | CAMK2B | 0.59 | -1.40 | 0.28 | 0.66 | 2.20 | 2.06 |
| Q13555 | CAMK2G | 0.44 | -0.76 | 0.44 | 0.73 | 1.60 | 1.49 |
| P27824 | CANX | 0.69 | -0.78 | 0.03 | -0.13 | 0.21 | 0.65 |
| P31323 | PRKAR2B | 0.41 | -0.69 | 0.71 | -0.89 | 0.09 | -0.19 |
| P16152 | CBR1 | 0.50 | -1.48 | 0.82 | 1.93 | 3.15 | 3.41 |
| P35222 | CTNNB1 | 0.05 | -0.05 | 1.38 | -1.10 | 1.96 | -1.05 |
| Q03135 | CAV1 | 0.34 | 0.68 | 1.50 | 1.98 | 0.93 | 1.30 |
| Q00610 | CLTC | 0.45 | -2.12 | 0.53 | 1.39 | 1.36 | 3.51 |
| P10909 | CLU | 0.56 | -1.53 | 0.48 | 1.16 | 2.05 | 2.69 |
| P23528 | CFL1 | 0.74 | -2.07 | 1.26 | -2.88 | 0.67 | -0.81 |
| Q14011 | CIRBP | 0.24 | 0.35 | 1.09 | -1.43 | 1.12 | -1.77 |
| P02452 | COL1A1 | 0.53 | -1.92 | 1.69 | -3.47 | 0.50 | -1.56 |
| P02461 | COL3A1 | 0.07 | 0.07 | 1.47 | -2.37 | 1.44 | -2.44 |
| P12109 | COL6A1 | 0.10 | -0.30 | 0.28 | 1.13 | 0.36 | 1.43 |
| P39060 | COL18A1 | 0.08 | -0.35 | 0.04 | 0.15 | 0.12 | 0.50 |
| P08123 | COL1A2 | 0.25 | -1.16 | 0.41 | -1.65 | 0.08 | -0.49 |
| P08572 | COL4A2 | 0.02 | 0.07 | 0.08 | -0.04 | 0.05 | -0.11 |
| P12110 | COL6A2 | 0.04 | 0.19 | 0.14 | 0.68 | 0.09 | 0.49 |
| P12111 | COL6A3 | 0.06 | -0.25 | 0.43 | -1.28 | 0.34 | -1.03 |
| O75367 | H2AFY | 3.45 | 2.87 | 1.38 | 1.37 | 1.56 | -1.50 |
| P12277 | CKB | 0.27 | -0.33 | 2.64 | 5.62 | 2.81 | 5.94 |
| P12532 | CKMT1A | 0.31 | -0.21 | 0.15 | -0.32 | 0.05 | -0.11 |
| P21291 | CSRP1 | 0.28 | 0.11 | 4.36 | 1.81 | 4.10 | 1.70 |
| P52943 | CRIP2 | 0.06 | 0.11 | 0.66 | -0.58 | 0.52 | -0.69 |
| P99999 | CYCS | 0.72 | -1.66 | 0.58 | -0.50 | 0.64 | 1.16 |
| Q14204 | DYNC1H1 | 0.02 | -0.09 | 0.49 | 1.54 | 0.59 | 1.63 |
| P60981 | DSTN | 0.35 | -0.90 | 0.08 | 0.17 | 0.91 | 1.08 |
| P09417 | QDPR | 1.39 | 1.97 | 1.86 | 1.68 | 0.17 | -0.29 |
| Q14194 | CRMP1 | 0.35 | -1.19 | 3.11 | -5.35 | 2.01 | -4.17 |
| Q16555 | DPYSL2 | 0.61 | -0.37 | 2.71 | -1.40 | 1.91 | -1.03 |
| Q14195 | DPYSL3 | 0.06 | -0.12 | 4.07 | -5.98 | 4.08 | -5.86 |
| Q9BPU6 | DPYSL5 | 1.03 | -0.67 | 4.69 | -4.43 | 4.60 | -3.75 |
| Q16643 | DBN1 | 0.25 | 0.37 | 1.11 | -2.82 | 1.22 | -3.19 |
| Q05193 | DNM1 | 0.37 | -0.24 | 3.65 | 3.80 | 3.82 | 4.04 |
| Q05639 | EEF1A2 | 0.64 | -0.98 | 0.51 | -0.83 | 0.11 | 0.15 |
| P49411 | TUFM | 0.40 | -0.21 | 2.17 | 0.63 | 2.81 | 0.84 |
| Q99962 | SH3GL2 | 1.04 | -1.72 | 0.13 | -0.31 | 1.09 | 1.42 |
| Q16658 | FSCN1 | 0.06 | 0.14 | 1.44 | -1.36 | 1.06 | -1.50 |
| P02671 | FGA | 0.36 | 2.08 | 0.99 | 3.19 | 0.20 | 1.11 |
| P02675 | FGB | 0.44 | 2.79 | 1.09 | 3.90 | 0.19 | 1.11 |
| P02679 | FGG | 0.46 | 2.59 | 1.14 | 3.80 | 0.23 | 1.21 |
| P02751 | FN1 | 0.58 | 2.38 | 3.07 | -3.23 | 1.78 | -5.61 |
| P04075 | ALDOA | 0.26 | -0.29 | 1.57 | 3.25 | 1.67 | 3.54 |
| P09972 | ALDOC | 0.39 | -0.53 | 1.64 | 3.43 | 1.82 | 3.96 |
| P14136 | GFAP | 1.13 | -1.54 | 1.55 | 4.66 | 2.04 | 6.20 |
| P06744 | GPI | 0.24 | -0.60 | 1.55 | 3.35 | 2.45 | 3.95 |
| P04406 | GAPDH | 0.06 | -0.10 | 1.17 | 1.52 | 1.22 | 1.62 |
| P09471 | GNAO1 | 0.47 | -0.54 | 0.08 | 0.23 | 0.30 | 0.77 |
| O43301 | HSPA12A | 0.63 | 0.53 | 4.09 | 3.86 | 3.55 | 3.32 |
| P0DMV9 | HSPA1B | 0.61 | 0.30 | 1.28 | 1.07 | 0.94 | 0.77 |
| P11142 | HSPA8 | 0.33 | 0.22 | 0.81 | 0.47 | 0.43 | 0.24 |
| P04792 | HSPB1 | 0.51 | -0.53 | 1.73 | 2.22 | 2.33 | 2.75 |
| P07900 | HSP90AA1 | 0.59 | -0.99 | 0.38 | 0.60 | 1.15 | 1.59 |
| P08238 | HSP90AB1 | 0.55 | -0.87 | 0.36 | 0.50 | 1.20 | 1.37 |
| P54652 | HSPA2 | 1.84 | 1.97 | 0.74 | 1.12 | 0.60 | -0.85 |
| P69905 | HBA1 | 0.39 | -0.88 | 1.11 | -2.02 | 1.00 | -1.14 |
| P68871 | HBB | 0.44 | -0.73 | 0.66 | 0.95 | 1.26 | 1.68 |
| P02042 | HBD | 0.49 | -1.21 | 1.30 | 1.84 | 2.09 | 3.05 |
| Q5SSJ5 | HP1BP3 | 1.18 | 1.41 | 3.97 | 2.46 | 1.01 | 1.04 |
| P09651 | HNRNPA1 | 0.27 | 0.60 | 0.89 | 2.19 | 0.78 | 1.59 |
| P51991 | HNRNPA3 | 0.20 | 0.28 | 0.85 | 1.79 | 0.77 | 1.50 |
| Q14103 | HNRNPD | 0.02 | 0.05 | 0.35 | 0.69 | 1.09 | 0.64 |
| P55795 | HNRNPH2 | 0.65 | 0.62 | 1.36 | 1.33 | 0.94 | 0.71 |
| P31942 | HNRNPH3 | 1.34 | 1.40 | 2.29 | 1.70 | 0.60 | 0.30 |
| P61978 | HNRNPK | 0.43 | 1.36 | 0.39 | -0.41 | 0.74 | -1.77 |
| P52272 | HNRNPM | 1.77 | 1.33 | 2.67 | 3.35 | 1.82 | 2.02 |
| O43390 | HNRNPR | 0.51 | 0.98 | 0.42 | 0.53 | 0.34 | -0.45 |
| Q00839 | HNRNPU | 2.24 | 1.27 | 1.52 | 1.58 | 0.23 | 0.31 |
| Q1KMD3 | HNRNPUL2 | 1.18 | 1.46 | 0.23 | 0.39 | 0.93 | -1.07 |
| P22626 | HNRNPA2B1 | 0.28 | 0.38 | 0.45 | 1.26 | 0.31 | 0.88 |
| P07910 | HNRNPC | 1.06 | 1.04 | 1.97 | 3.07 | 1.50 | 2.04 |
| P19367 | HK1 | 0.42 | -0.64 | 0.24 | 0.30 | 0.76 | 0.93 |
| P12314 | FCGR1A | 0.04 | -0.13 | 0.61 | 0.96 | 0.82 | 1.09 |
| P07305 | H1F0 | 1.62 | 1.46 | 2.04 | 2.51 | 0.96 | 1.05 |
| P16403 | HIST1H1C | 1.66 | 2.69 | 1.40 | 1.79 | 0.59 | -0.90 |
| P16401 | HIST1H1B | 0.97 | 0.61 | 1.70 | -3.34 | 1.98 | -3.95 |
| Q92522 | H1FX | 0.64 | 1.30 | 1.38 | -2.25 | 3.05 | -3.56 |
| Q99878 | HIST1H2AJ | 0.25 | -1.69 | 0.57 | -2.45 | 0.15 | -0.76 |
| Q99880 | HIST1H2BL | 1.65 | 1.96 | 2.16 | 4.07 | 1.23 | 2.10 |
| Q16778 | HIST2H2BE | 0.98 | 2.66 | 2.91 | 4.98 | 1.06 | 2.32 |
| Q71DI3 | HIST2H3A | 1.32 | 1.80 | 1.71 | 1.62 | 0.10 | -0.18 |
| P62805 | HIST1H4A | 1.67 | 1.82 | 1.53 | 1.40 | 0.50 | -0.43 |
| P01876 | IGHA1 | 0.09 | -0.35 | 1.12 | 2.61 | 2.45 | 2.96 |
| P0DOX5 | IGHG1 | 0.11 | 0.39 | 0.24 | -0.76 | 0.57 | -1.14 |
| P01859 | IGHG2 | 0.17 | -0.39 | 0.81 | -1.10 | 0.46 | -0.71 |
| P01860 | IGHG3 | 0.20 | -0.71 | 0.40 | -1.27 | 0.26 | -0.56 |
| P01834 | IGKC | 0.24 | -0.72 | 1.05 | -1.76 | 0.77 | -1.03 |
| P0DOX8 | IGLL5 | 0.20 | 0.71 | 0.20 | -0.83 | 0.48 | -1.54 |
| Q14974 | KPNB1 | 0.69 | -0.80 | 0.37 | -0.45 | 0.36 | 0.35 |
| P48735 | IDH2 | 0.26 | -0.29 | 1.29 | 1.77 | 1.37 | 2.06 |
| Q07666 | KHDRBS1 | 0.51 | 0.63 | 1.12 | -0.93 | 1.47 | -1.56 |
| O75525 | KHDRBS3 | 0.22 | 0.29 | 1.13 | -2.00 | 1.19 | -2.29 |
| P20700 | LMNB1 | 0.86 | 0.64 | 4.77 | -4.20 | 4.64 | -4.84 |
| Q03252 | LMNB2 | 0.88 | 1.13 | 0.58 | 0.94 | 0.08 | -0.19 |
| P42167 | TMPO | 0.18 | -0.24 | 1.92 | -2.66 | 1.60 | -2.43 |
| P24043 | LAMA2 | 0.39 | 1.70 | 1.38 | 2.11 | 0.11 | 0.42 |
| O15230 | LAMA5 | 0.04 | -0.15 | 3.77 | 4.12 | 2.22 | 4.26 |
| P55268 | LAMB2 | 0.21 | 0.46 | 1.74 | 4.49 | 1.42 | 4.04 |
| P11047 | LAMC1 | 0.01 | 0.02 | 0.56 | 0.54 | 0.25 | 0.52 |
| O43813 | LANCL1 | 1.09 | -2.11 | 0.23 | 0.45 | 1.96 | 2.56 |
| P40926 | MDH2 | 0.35 | -0.56 | 0.99 | 1.12 | 3.14 | 1.68 |
| P43243 | MATR3 | 0.60 | 1.05 | 0.37 | 0.66 | 0.31 | -0.39 |
| P51608 | MECP2 | 1.99 | 1.82 | 0.80 | 1.02 | 0.52 | -0.79 |
| P78559 | MAP1A | 0.74 | 1.01 | 3.27 | 2.80 | 1.85 | 1.79 |
| P46821 | MAP1B | 0.44 | 0.48 | 3.13 | -1.86 | 2.90 | -2.33 |
| P11137 | MAP2 | 0.41 | -1.18 | 0.43 | -1.30 | 0.04 | -0.12 |
| P27816 | MAP4 | 0.04 | 0.06 | 1.43 | -1.20 | 1.39 | -1.26 |
| P10636 | MAPT | 0.04 | -0.08 | 0.03 | -0.06 | 0.01 | 0.02 |
| P28482 | MAPK1 | 0.73 | -1.11 | 0.37 | 0.51 | 3.81 | 1.61 |
| O95819 | MAP4K4 | 0.30 | 0.47 | 0.66 | -1.71 | 0.97 | -2.18 |
| Q9H8L6 | MMRN2 | 0.23 | -0.50 | 1.60 | -2.66 | 2.18 | -2.16 |
| P02686 | MBP | 1.17 | 1.83 | 2.51 | 7.63 | 2.10 | 5.81 |
| P02689 | PMP2 | 1.05 | 1.65 | 1.75 | 2.14 | 0.30 | 0.49 |
| P60201 | PLP1 | 1.48 | 1.55 | 2.10 | 5.45 | 1.54 | 3.90 |
| Q16653 | MOG | 0.89 | 1.48 | 2.65 | 2.05 | 0.38 | 0.57 |
| P60660 | MYL6 | 0.15 | 0.41 | 0.95 | 1.29 | 0.45 | 0.89 |
| P19105 | MYL12A | 1.10 | 2.29 | 1.16 | 0.79 | 0.90 | -1.49 |
| P24844 | MYL9 | 1.02 | 2.03 | 1.38 | 1.46 | 0.25 | -0.57 |
| P35580 | MYH10 | 0.00 | 0.00 | 0.78 | -1.69 | 1.01 | -1.68 |
| P35749 | MYH11 | 0.25 | 1.28 | 3.40 | 5.32 | 1.20 | 4.04 |
| P35579 | MYH9 | 0.39 | 0.78 | 1.58 | 3.50 | 1.31 | 2.72 |
| P29966 | MARCKS | 0.27 | 0.31 | 0.57 | -1.34 | 0.73 | -1.65 |
| P13591 | NCAM1 | 0.31 | 0.39 | 2.28 | -2.99 | 3.03 | -3.38 |
| P12036 | NEFH | 1.23 | 1.64 | 4.03 | 6.27 | 3.02 | 4.63 |
| P07196 | NEFL | 1.47 | 1.31 | 5.04 | 8.20 | 4.26 | 6.89 |
| P07197 | NEFM | 1.74 | 1.40 | 3.00 | 8.00 | 2.61 | 6.60 |
| P17677 | GAP43 | 0.15 | -0.25 | 1.11 | -1.99 | 1.01 | -1.73 |
| P51674 | GPM6A | 0.50 | 0.49 | 0.73 | -1.80 | 0.94 | -2.29 |
| O43602 | DCX | 0.21 | -0.32 | 3.53 | -5.47 | 3.06 | -5.15 |
| P14543 | NID1 | 0.17 | 0.65 | 0.95 | 1.28 | 0.20 | 0.63 |
| Q14112 | NID2 | 0.43 | 1.37 | 0.04 | -0.08 | 0.58 | -1.45 |
| Q15233 | NONO | 0.08 | -0.37 | 1.51 | -4.20 | 1.56 | -3.83 |
| Q14980 | NUMA1 | 3.17 | 3.64 | 2.58 | 2.22 | 2.52 | -1.42 |
| P19338 | NCL | 0.32 | 0.95 | 0.50 | 1.15 | 0.06 | 0.19 |
| Q8WXF1 | PSPC1 | 0.15 | 0.22 | 1.20 | -2.66 | 1.40 | -2.88 |
| P62937 | PPIA | 1.02 | -1.92 | 0.28 | 0.69 | 1.24 | 2.62 |
| Q06830 | PRDX1 | 0.12 | -0.15 | 0.05 | 0.11 | 0.12 | 0.26 |
| P32119 | PRDX2 | 0.38 | 0.40 | 0.54 | -0.52 | 1.21 | -0.92 |
| P30086 | PEBP1 | 0.09 | 0.08 | 2.21 | -2.88 | 2.38 | -2.95 |
| P18669 | PGAM1 | 1.12 | -3.40 | 0.02 | 0.07 | 2.54 | 3.48 |
| Q15149 | PLEC | 0.74 | -2.43 | 0.93 | 3.05 | 2.49 | 5.48 |
| P09874 | PARP1 | 1.72 | 2.95 | 3.12 | 3.11 | 0.08 | 0.17 |
| Q15366 | PCBP2 | 0.71 | 1.50 | 0.04 | -0.07 | 0.92 | -1.57 |
| Q6NZI2 | PTRF | 0.44 | 0.64 | 3.27 | 4.33 | 2.40 | 3.70 |
| Q6P2Q9 | PRPF8 | 0.26 | 0.19 | 2.26 | -1.03 | 1.86 | -1.22 |
| P02545 | LMNA | 1.27 | 0.88 | 2.05 | 1.61 | 0.75 | 0.74 |
| Q92841 | DDX17 | 0.76 | 0.90 | 1.69 | -1.34 | 2.39 | -2.24 |
| P35080 | PFN2 | 0.96 | -0.61 | 0.06 | 0.14 | 0.47 | 0.75 |
| P41222 | PTGDS | 0.03 | -0.10 | 0.86 | 0.78 | 0.47 | 0.88 |
| P21980 | TGM2 | 0.13 | -0.36 | 0.21 | -0.67 | 0.11 | -0.31 |
| P22061 | PCMT1 | 0.42 | -0.70 | 0.50 | 0.79 | 1.65 | 1.49 |
| Q5VTE0 | EEF1A1P5 | 0.40 | -1.14 | 0.93 | -2.09 | 0.91 | -0.95 |
| O00764 | PDXK | 0.66 | -2.17 | 0.51 | 1.23 | 2.36 | 3.40 |
| P14618 | PKM | 0.99 | -1.17 | 2.24 | 4.92 | 2.71 | 6.09 |
| P31150 | GDI1 | 1.13 | -1.50 | 0.56 | -1.37 | 0.04 | 0.13 |
| P35637 | FUS | 0.38 | 0.68 | 1.13 | -2.24 | 1.25 | -2.93 |
| P63208 | SKP1 | 0.42 | 0.59 | 1.02 | -0.75 | 1.14 | -1.34 |
| Q15019 | 2-Sep | 0.23 | -0.60 | 0.37 | 0.55 | 0.76 | 1.15 |
| Q16181 | 7-Sep | 0.31 | -1.14 | 0.33 | -0.72 | 0.13 | 0.41 |
| Q13501 | SQSTM1 | 1.13 | -2.66 | 0.07 | -0.18 | 1.03 | 2.47 |
| O15075 | DCLK1 | 0.30 | -0.50 | 0.87 | -2.64 | 0.75 | -2.14 |
| P30153 | PPP2R1A | 0.64 | -2.46 | 0.05 | 0.16 | 1.22 | 2.61 |
| P50454 | SERPINH1 | 0.63 | 0.38 | 2.09 | -3.68 | 2.30 | -4.07 |
| O95810 | SDPR | 0.03 | -0.09 | 0.07 | 0.16 | 0.24 | 0.25 |
| P11166 | SLC2A1 | 0.74 | 0.67 | 1.05 | -2.69 | 1.29 | -3.36 |
| Q13813 | SPTAN1 | 0.57 | -0.56 | 0.38 | 0.48 | 1.04 | 1.04 |
| Q01082 | SPTBN1 | 0.60 | -0.64 | 0.38 | 0.48 | 1.11 | 1.12 |
| P23246 | SFPQ | 0.06 | 0.21 | 2.05 | -5.29 | 2.31 | -5.50 |
| P17600 | SYN1 | 0.46 | -0.75 | 1.21 | 2.78 | 1.72 | 3.52 |
| Q92777 | SYN2 | 1.01 | -1.13 | 0.90 | 1.32 | 1.87 | 2.45 |
| P61764 | STXBP1 | 0.31 | -0.41 | 1.22 | 2.09 | 1.40 | 2.50 |
| O00560 | SDCBP | 0.87 | -0.68 | 0.49 | -0.39 | 0.25 | 0.29 |
| P78371 | CCT2 | 0.06 | 0.14 | 0.32 | -0.43 | 0.50 | -0.57 |
| P48643 | CCT5 | 0.27 | -0.41 | 0.84 | -0.79 | 0.32 | -0.38 |
| Q9Y490 | TLN1 | 0.33 | -1.19 | 0.52 | 1.36 | 2.15 | 2.55 |
| Q13148 | TARDBP | 0.82 | 1.12 | 0.82 | 0.91 | 0.33 | -0.21 |
| Q92752 | TNR | 0.66 | 1.54 | 0.71 | 1.27 | 0.12 | -0.28 |
| Q86V81 | ALYREF | 0.20 | 0.38 | 1.33 | -2.64 | 1.32 | -3.02 |
| Q13263 | TRIM28 | 1.46 | 2.73 | 1.11 | 1.83 | 0.58 | -0.90 |
| Q00577 | PURA | 0.10 | 0.13 | 1.37 | 1.55 | 1.67 | 1.41 |
| P29401 | TKT | 0.70 | -0.48 | 3.67 | 2.24 | 3.67 | 2.72 |
| P40939 | HADHA | 0.17 | 0.50 | 0.90 | 1.77 | 0.82 | 1.27 |
| P55084 | HADHB | 0.12 | -0.32 | 0.60 | 1.14 | 0.90 | 1.46 |
| Q9NZR1 | TMOD2 | 0.21 | -0.50 | 0.23 | 0.39 | 0.69 | 0.89 |
| P68366 | TUBA4A | 0.33 | 0.30 | 3.01 | 4.61 | 2.79 | 4.32 |
| P07437 | TUBB | 0.70 | 0.37 | 2.95 | -1.42 | 4.00 | -1.78 |
| Q13509 | TUBB3 | 0.82 | 0.88 | 0.36 | 1.21 | 0.08 | 0.33 |
| P04350 | TUBB4A | 2.02 | 1.19 | 1.98 | 4.24 | 1.45 | 3.05 |
| O94811 | TPPP | 0.88 | -0.38 | 1.38 | 2.76 | 1.57 | 3.14 |
| Q9GZM7 | TINAGL1 | 0.20 | 0.46 | 2.10 | 2.83 | 1.39 | 2.37 |
| P09936 | UCHL1 | 1.05 | -3.48 | 0.03 | -0.10 | 1.55 | 3.39 |
| P62987 | UBA52 | 1.46 | -3.02 | 1.33 | -1.50 | 0.90 | 1.51 |
| P22314 | UBA1 | 0.54 | -2.43 | 0.21 | 0.81 | 1.99 | 3.24 |
| O95670 | ATP6V1G2 | 1.06 | -1.05 | 0.61 | -0.42 | 1.33 | 0.63 |
| P13611 | VCAN | 1.18 | 2.02 | 2.44 | 2.73 | 0.47 | 0.71 |
| P46459 | NSF | 0.13 | -0.12 | 3.29 | 4.08 | 3.29 | 4.20 |
| P08670 | VIM | 0.38 | 0.33 | 1.56 | 0.89 | 1.33 | 0.57 |
| P18206 | VCL | 0.02 | -0.10 | 0.34 | 0.75 | 0.33 | 0.86 |
| P45880 | VDAC2 | 0.93 | -1.62 | 0.95 | -1.47 | 0.14 | 0.15 |
| Q13303 | KCNAB2 | 0.74 | 0.82 | 0.98 | 0.74 | 0.06 | -0.08 |
| P13010 | XRCC5 | 0.01 | 0.02 | 1.19 | 2.28 | 1.68 | 2.26 |
| P0DOX7 | *** | 0.15 | 0.65 | 1.44 | -2.64 | 1.58 | -3.29 |
| P0DP25 | CALM3 | 0.03 | -0.04 | 0.66 | 1.49 | 0.64 | 1.53 |
| ***Protein ID does not map to a unique gene. | | | | | | | |
