# Supplementary material for: Characterization of the Tau Interactome in Human Brain Reveals Isoform-Dependent Interaction with 14-3-3 Family Proteins
Source: eNeuro. 2023 Mar 21;10(3):ENEURO.0503-22.2023. doi: 10.1523/ENEURO.0503-22.2023 (PMC10035768; doi:10.1523/ENEURO.0503-22.2023)
Supplement: Extended Data Table 4-1 — Table of all gene ontology term enrichments (AD). Download Table 4-1, DOC file. [file enu-eN-NWR-0503-22-s02.doc]

**Table 4-1^b^. Table of all gene ontology term enrichments (AD)**

| Ontology | Term | Total | Expected | FE | P-value | FDR |
| --- | --- | --- | --- | --- | --- | --- |
| BP | neurofilament bundle assembly (GO:0033693) | 3 | 0.01 | > 100 | 6.48E-07 | 3.28E-04 |
| BP | axon target recognition (GO:0007412) | 2 | 0.01 | > 100 | 1.03E-04 | 1.38E-02 |
| BP | postsynaptic intermediate filament cytoskeleton organization (GO:0099185) | 2 | 0.01 | > 100 | 1.03E-04 | 1.37E-02 |
| BP | isocitrate metabolic process (GO:0006102) | 2 | 0.02 | > 100 | 2.87E-04 | 3.10E-02 |
| BP | fructose 1,6-bisphosphate metabolic process (GO:0030388) | 3 | 0.03 | > 100 | 7.03E-06 | 1.78E-03 |
| BP | aldehyde biosynthetic process (GO:0046184) | 2 | 0.03 | 76.82 | 4.59E-04 | 4.49E-02 |
| BP | anterograde axonal protein transport (GO:0099641) | 2 | 0.03 | 76.82 | 4.59E-04 | 4.47E-02 |
| BP | neurofilament cytoskeleton organization (GO:0060052) | 2 | 0.03 | 68.29 | 5.59E-04 | 5.07E-02 |
| BP | oxaloacetate metabolic process (GO:0006107) | 2 | 0.03 | 68.29 | 5.59E-04 | 5.04E-02 |
| BP | skeletal myofibril assembly (GO:0014866) | 2 | 0.03 | 68.29 | 5.59E-04 | 5.01E-02 |
| BP | synaptic vesicle clustering (GO:0097091) | 2 | 0.03 | 68.29 | 5.59E-04 | 4.98E-02 |
| BP | myelin maintenance (GO:0043217) | 3 | 0.06 | 54.23 | 3.57E-05 | 5.84E-03 |
| BP | glycolytic process (GO:0006096) | 6 | 0.14 | 43.9 | 1.02E-08 | 5.34E-05 |
| BP | platelet aggregation (GO:0070527) | 6 | 0.14 | 42.88 | 1.16E-08 | 3.64E-05 |
| BP | substantia nigra development (GO:0021762) | 5 | 0.15 | 32.69 | 7.12E-07 | 3.49E-04 |
| BP | tricarboxylic acid cycle (GO:0006099) | 3 | 0.1 | 29.74 | 1.82E-04 | 2.16E-02 |
| BP | neuron projection regeneration (GO:0031102) | 3 | 0.1 | 29.74 | 1.82E-04 | 2.14E-02 |
| BP | NADH metabolic process (GO:0006734) | 3 | 0.11 | 27.11 | 2.34E-04 | 2.62E-02 |
| BP | astrocyte development (GO:0014002) | 3 | 0.11 | 26.34 | 2.54E-04 | 2.82E-02 |
| BP | cortical actin cytoskeleton organization (GO:0030866) | 3 | 0.13 | 23.64 | 3.42E-04 | 3.62E-02 |
| BP | cell-substrate junction assembly (GO:0007044) | 3 | 0.13 | 23.05 | 3.67E-04 | 3.83E-02 |
| BP | gluconeogenesis (GO:0006094) | 3 | 0.14 | 21.95 | 4.20E-04 | 4.25E-02 |
| BP | oligodendrocyte development (GO:0014003) | 3 | 0.14 | 21.44 | 4.48E-04 | 4.42E-02 |
| BP | calcium-ion regulated exocytosis (GO:0017156) | 3 | 0.15 | 20.49 | 5.08E-04 | 4.72E-02 |
| BP | regulation of synaptic plasticity (GO:0048167) | 9 | 0.63 | 14.26 | 1.87E-08 | 4.89E-05 |
| BP | regulation of neurotransmitter transport (GO:0051588) | 4 | 0.35 | 11.38 | 4.83E-04 | 4.56E-02 |
| BP | negative regulation of protein-containing complex assembly (GO:0031333) | 5 | 0.47 | 10.74 | 1.19E-04 | 1.52E-02 |
| BP | anatomical structure homeostasis (GO:0060249) | 6 | 0.99 | 6.09 | 4.95E-04 | 4.65E-02 |
| BP | microtubule cytoskeleton organization (GO:0000226) | 10 | 1.78 | 5.62 | 1.17E-05 | 2.62E-03 |
| BP | negative regulation of protein modification process (GO:0031400) | 9 | 1.64 | 5.5 | 3.92E-05 | 6.35E-03 |
| BP | chemical synaptic transmission (GO:0007268) | 7 | 1.35 | 5.2 | 4.22E-04 | 4.24E-02 |
| BP | negative regulation of phosphate metabolic process (GO:0045936) | 7 | 1.37 | 5.1 | 4.72E-04 | 4.51E-02 |
| BP | protein-containing complex assembly (GO:0065003) | 13 | 4.13 | 3.15 | 2.10E-04 | 2.41E-02 |
| BP | response to chemical (GO:0042221) | 26 | 13.21 | 1.97 | 3.06E-04 | 3.28E-02 |
| CC | postsynaptic intermediate filament cytoskeleton (GO:0099160) | 3 | 0.01 | > 100 | 1.13E-06 | 6.80E-05 |
| CC | laminin-11 complex (GO:0043260) | 2 | 0.01 | > 100 | 1.03E-04 | 3.51E-03 |
| CC | neurofibrillary tangle (GO:0097418) | 3 | 0.02 | > 100 | 1.81E-06 | 9.71E-05 |
| CC | internode region of axon (GO:0033269) | 2 | 0.01 | > 100 | 1.54E-04 | 4.70E-03 |
| CC | calcium- and calmodulin-dependent protein kinase complex (GO:0005954) | 2 | 0.02 | > 100 | 2.15E-04 | 6.11E-03 |
| CC | neurofilament (GO:0005883) | 3 | 0.04 | 83.81 | 1.16E-05 | 4.73E-04 |
| CC | pseudopodium (GO:0031143) | 3 | 0.06 | 51.22 | 4.16E-05 | 1.55E-03 |
| CC | synaptic cleft (GO:0043083) | 2 | 0.06 | 32.35 | 2.09E-03 | 4.80E-02 |
| CC | myelin sheath (GO:0043209) | 5 | 0.16 | 32.01 | 7.85E-07 | 5.53E-05 |
| CC | axon cytoplasm (GO:1904115) | 4 | 0.2 | 19.83 | 6.26E-05 | 2.28E-03 |
| CC | ficolin-1-rich granule lumen (GO:1904813) | 7 | 0.4 | 17.35 | 2.23E-07 | 1.68E-05 |
| CC | neuromuscular junction (GO:0031594) | 4 | 0.24 | 16.39 | 1.26E-04 | 4.10E-03 |
| CC | Schaffer collateral - CA1 synapse (GO:0098685) | 4 | 0.25 | 15.96 | 1.39E-04 | 4.45E-03 |
| CC | platelet alpha granule lumen (GO:0031093) | 3 | 0.22 | 13.76 | 1.53E-03 | 3.67E-02 |
| CC | Z disc (GO:0030018) | 5 | 0.42 | 11.82 | 7.72E-05 | 2.72E-03 |
| CC | melanosome (GO:0042470) | 4 | 0.36 | 11.07 | 5.33E-04 | 1.43E-02 |
| CC | focal adhesion (GO:0005925) | 12 | 1.38 | 8.72 | 1.38E-08 | 1.34E-06 |
| CC | postsynaptic density (GO:0014069) | 7 | 1.06 | 6.6 | 1.01E-04 | 3.49E-03 |
| CC | extracellular exosome (GO:0070062) | 44 | 6.83 | 6.44 | 1.64E-27 | 3.36E-24 |
| CC | dendrite (GO:0030425) | 10 | 2.05 | 4.89 | 3.79E-05 | 1.46E-03 |
| CC | microtubule (GO:0005874) | 7 | 1.53 | 4.57 | 8.93E-04 | 2.34E-02 |
| CC | neuronal cell body (GO:0043025) | 7 | 1.64 | 4.26 | 1.33E-03 | 3.35E-02 |
| CC | cell-cell junction (GO:0005911) | 7 | 1.65 | 4.23 | 1.37E-03 | 3.42E-02 |
| CC | actin cytoskeleton (GO:0015629) | 7 | 1.66 | 4.22 | 1.40E-03 | 3.42E-02 |
| CC | mitochondrion (GO:0005739) | 19 | 5.44 | 3.49 | 1.06E-06 | 6.57E-05 |
| CC | vacuole (GO:0005773) | 9 | 2.75 | 3.27 | 1.68E-03 | 4.00E-02 |
| CC | cytosol (GO:0005829) | 50 | 17.76 | 2.82 | 3.44E-16 | 7.80E-14 |
| CC | integral component of membrane (GO:0016021) | 3 | 18.87 | 0.16 | 1.62E-06 | 8.92E-05 |
| MF | fructose-bisphosphate aldolase activity (GO:0004332) | 2 | 0.01 | > 100 | 1.03E-04 | 1.35E-02 |
| MF | protein kinase C inhibitor activity (GO:0008426) | 2 | 0.01 | > 100 | 1.03E-04 | 1.31E-02 |
| MF | structural constituent of postsynaptic intermediate filament cytoskeleton (GO:0099184) | 2 | 0.01 | > 100 | 1.03E-04 | 1.28E-02 |
| MF | cytoskeletal protein-membrane anchor activity (GO:0106006) | 2 | 0.01 | > 100 | 1.54E-04 | 1.74E-02 |
| MF | low-density lipoprotein particle receptor binding (GO:0050750) | 3 | 0.08 | 36.88 | 1.01E-04 | 1.36E-02 |
| MF | glutamate receptor binding (GO:0035254) | 3 | 0.14 | 21.95 | 4.20E-04 | 4.35E-02 |
| MF | structural constituent of muscle (GO:0008307) | 3 | 0.14 | 20.95 | 4.78E-04 | 4.75E-02 |
| MF | integrin binding (GO:0005178) | 7 | 0.52 | 13.53 | 1.11E-06 | 3.67E-04 |
| MF | calmodulin binding (GO:0005516) | 6 | 0.65 | 9.17 | 5.65E-05 | 8.79E-03 |
| MF | actin filament binding (GO:0051015) | 6 | 0.72 | 8.31 | 9.64E-05 | 1.33E-02 |
| MF | cadherin binding (GO:0045296) | 8 | 1.06 | 7.56 | 1.20E-05 | 3.13E-03 |
| MF | microtubule binding (GO:0008017) | 6 | 0.89 | 6.73 | 2.93E-04 | 3.11E-02 |
| MF | ubiquitin protein ligase binding (GO:0031625) | 6 | 0.98 | 6.13 | 4.79E-04 | 4.67E-02 |
| MF | protein domain specific binding (GO:0019904) | 13 | 2.24 | 5.8 | 3.38E-07 | 1.29E-04 |
| MF | protein kinase binding (GO:0019901) | 11 | 2.3 | 4.77 | 1.83E-05 | 3.64E-03 |
| MF | protein homodimerization activity (GO:0042803) | 11 | 2.32 | 4.75 | 1.93E-05 | 3.69E-03 |
| MF | RNA binding (GO:0003723) | 22 | 5.42 | 4.06 | 7.65E-09 | 6.34E-06 |
| MF | ATP binding (GO:0005524) | 15 | 4.86 | 3.08 | 7.83E-05 | 1.15E-02 |
