# Supplementary material for: Characterization of the Tau Interactome in Human Brain Reveals Isoform-Dependent Interaction with 14-3-3 Family Proteins
Source: eNeuro. 2023 Mar 21;10(3):ENEURO.0503-22.2023. doi: 10.1523/ENEURO.0503-22.2023 (PMC10035768; doi:10.1523/ENEURO.0503-22.2023)
Supplement: Extended Data Table 4-2 — Table of all gene ontology term enrichments (Fetal). Download Table 4-2, DOC file. [file enu-eN-NWR-0503-22-s03.doc]

**Table 4-2^b^. Table of all gene ontology term enrichments (Fetal)**

| **Ontology** | **Term** | **Total** | **Expected** | **FE** | **P-value** | **FDR** |
| --- | --- | --- | --- | --- | --- | --- |
| BP | positive regulation of axon extension (GO:0045773) | 3 | 0.05 | 58.66 | 2.22E-05 | 4.98E-02 |
| BP | positive regulation of response to biotic stimulus (GO:0002833) | 5 | 0.25 | 20.17 | 4.78E-06 | 3.75E-02 |
| BP | negative regulation of biological process (GO:0048519) | 19 | 6.97 | 2.73 | 1.60E-06 | 2.51E-02 |
| MF | structural molecule activity (GO:0005198) | 9 | 1.04 | 8.62 | 5.02E-07 | 1.25E-03 |
| MF | protein-containing complex binding (GO:0044877) | 10 | 1.72 | 5.81 | 3.50E-06 | 5.80E-03 |
| MF | RNA binding (GO:0003723) | 12 | 2.18 | 5.49 | 4.44E-07 | 2.21E-03 |
| CC | paraspeckles (GO:0042382) | 3 | 0.01 | > 100 | 3.28E-07 | 2.24E-04 |
| CC | nuclear matrix (GO:0016363) | 6 | 0.17 | 36.31 | 1.63E-08 | 3.33E-05 |
| CC | growth cone (GO:0030426) | 5 | 0.22 | 22.56 | 2.81E-06 | 1.43E-03 |
| CC | cortical cytoskeleton (GO:0030863) | 3 | 0.15 | 20.61 | 4.37E-04 | 4.47E-02 |
| CC | nuclear speck (GO:0016607) | 6 | 0.55 | 10.92 | 1.52E-05 | 3.88E-03 |
| CC | collagen-containing extracellular matrix (GO:0062023) | 5 | 0.57 | 8.79 | 2.35E-04 | 2.82E-02 |
| CC | cell body (GO:0044297) | 6 | 0.75 | 7.97 | 8.64E-05 | 1.36E-02 |
| CC | supramolecular fiber (GO:0099512) | 8 | 1.37 | 5.83 | 4.25E-05 | 8.68E-03 |
