# Supplementary figures and images for: Characterization of the Tau Interactome in Human Brain Reveals Isoform-Dependent Interaction with 14-3-3 Family Proteins
Source: eNeuro. 2023 Mar 21;10(3):ENEURO.0503-22.2023. doi: 10.1523/ENEURO.0503-22.2023 (PMC10035768; doi:10.1523/ENEURO.0503-22.2023)

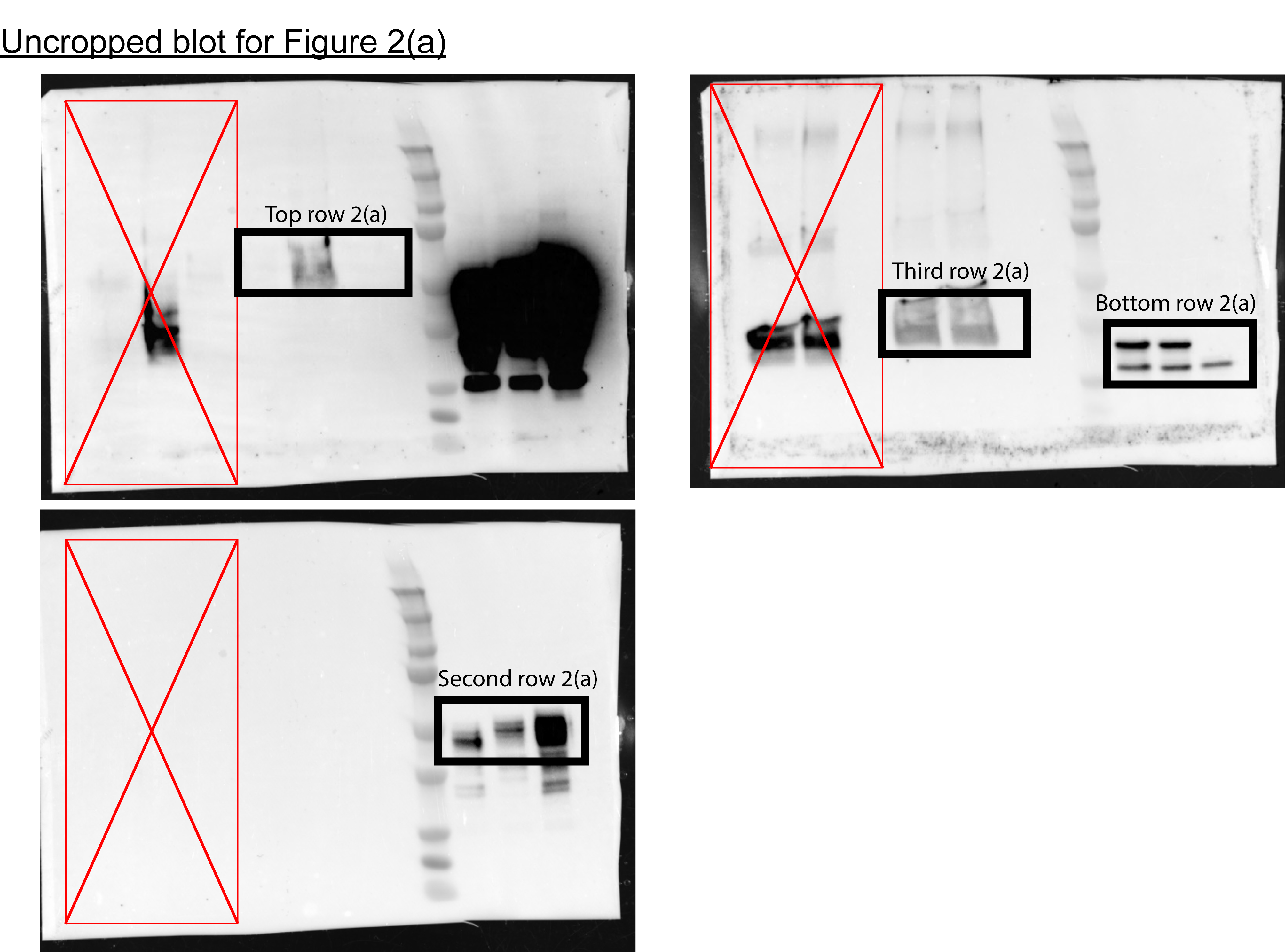

Supplement: Extended Data Figure 2-1 — Uncropped western blot images for Figure 2A. Red boxes indicate irrelevant bands from unrelated experiment. Boxes indicate bands shown in Figure 2A. Additional bands in top left, and bottom left, right side represent tau degradation products. Download Figure 2-1, TIF file. [file enu-eN-NWR-0503-22-s04.tif]

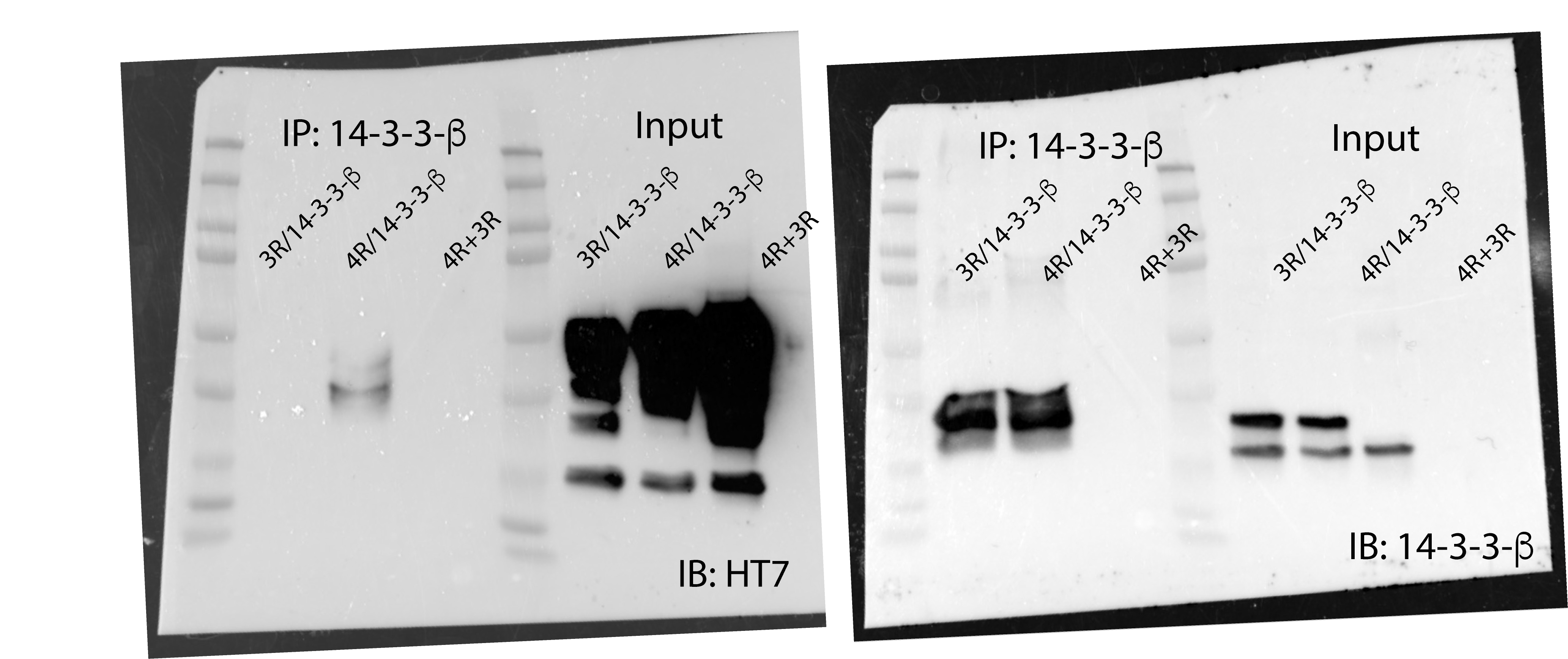

Supplement: Extended Data Figure 2-2 — Replication of co-IP in Figure 2A, with uncropped blots. Download Figure 2-2, TIF file. [file enu-eN-NWR-0503-22-s05.tif]

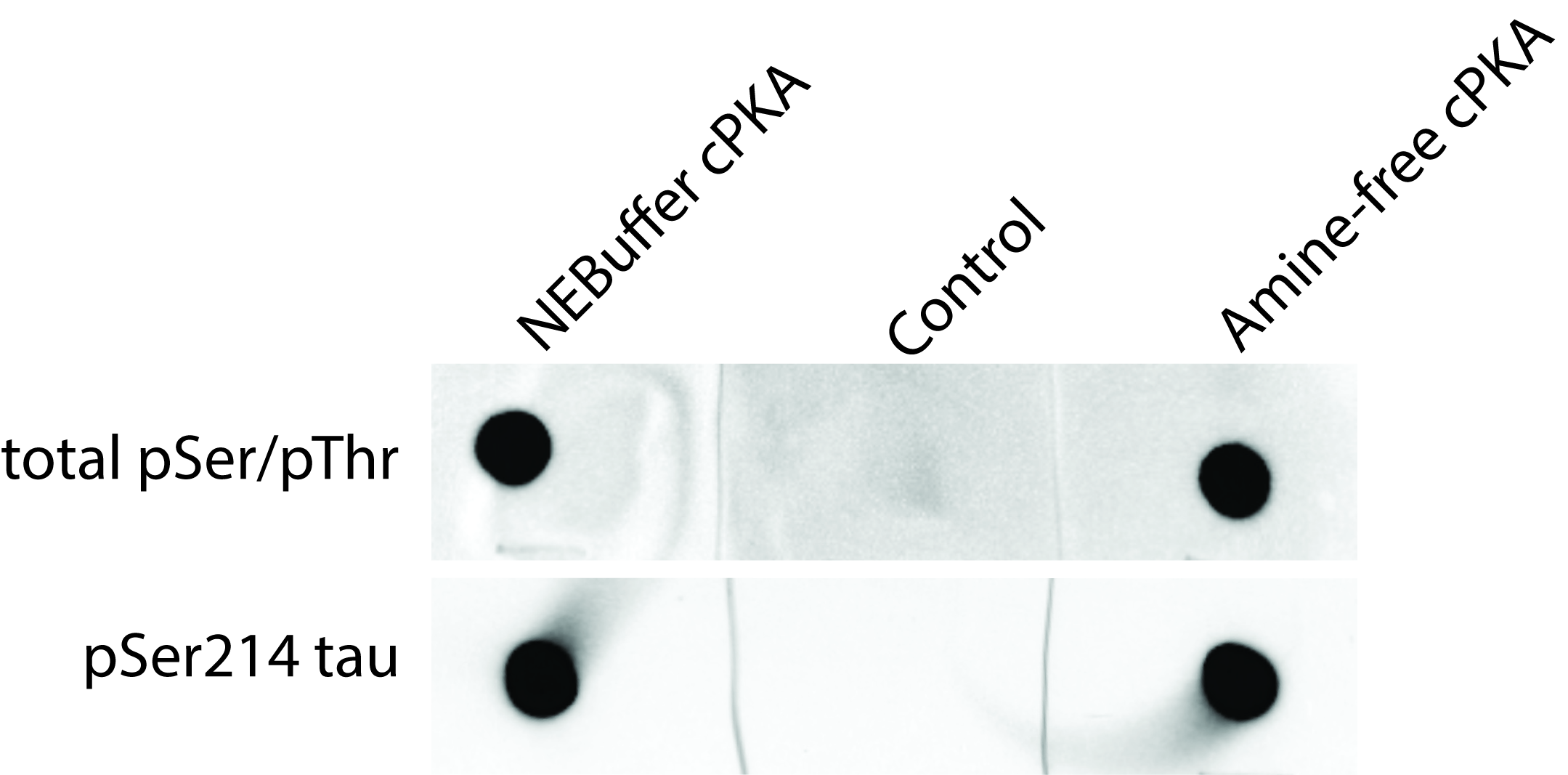

Supplement: Extended Data Figure 2-3 — Validation of tau phosphorylation by PKA for mass photometry. We added 1 μg of the phosphorylated, recombinant tau to a nitrocellulose membrane and probed with anti-tau Ser214 and total pSer/pThr antibodies as indicated. Control, unphosphorylated tau. Download Figure 2-3, TIF file. [file enu-eN-NWR-0503-22-s06.tif]

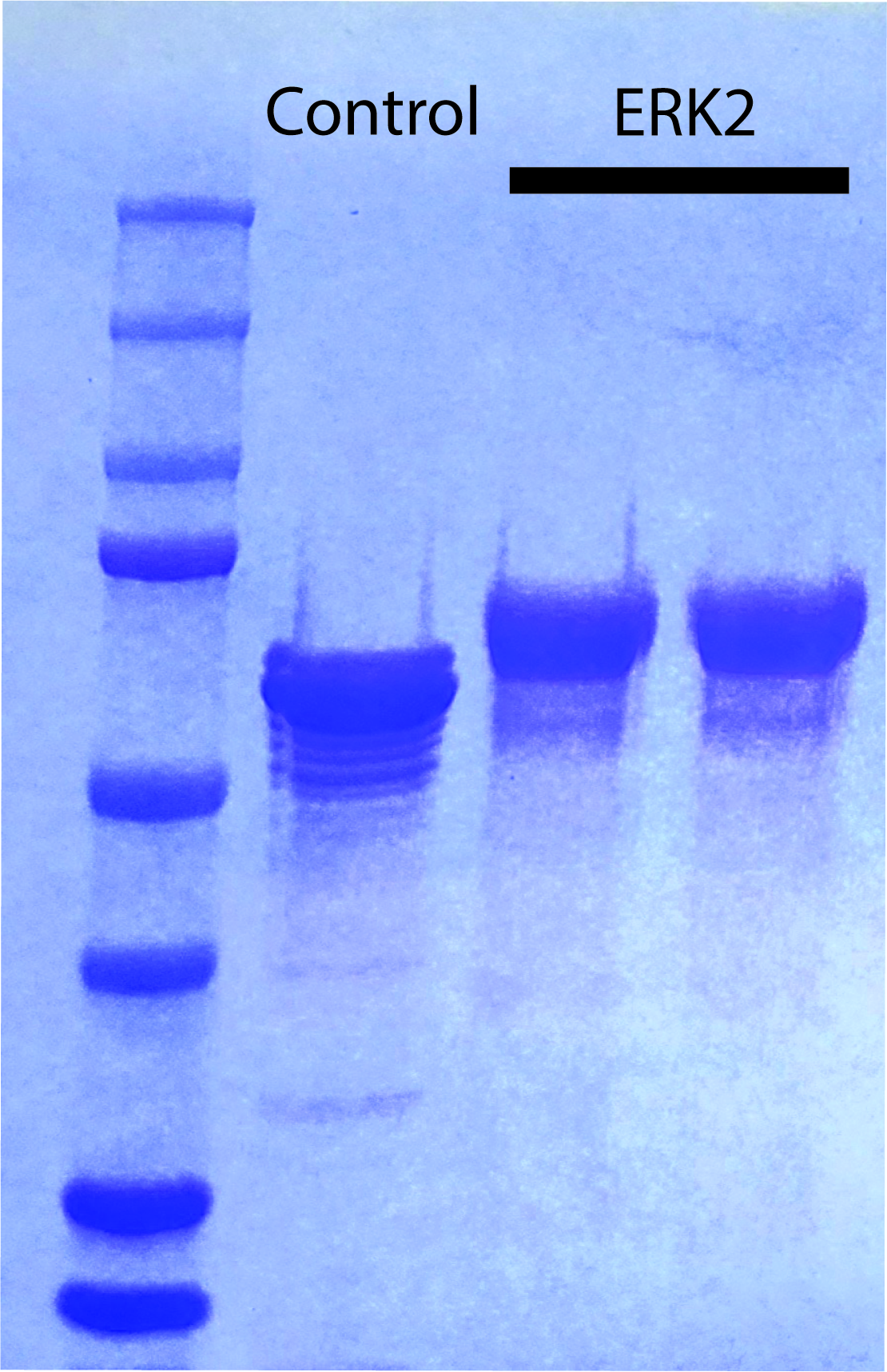

Supplement: Extended Data Figure 2-4 — Mass photometry histogram of ptau only, as in Figure 2B. Download Figure 2-4, TIF file. [file enu-eN-NWR-0503-22-s07.tif]
